# Rib‐Reinforced Ultralight and Ultra‐Strong Shell Lattices

**DOI:** 10.1002/advs.202518357

**Published:** 2026-01-21

**Authors:** Winston Wai Shing Ma, Lei Zhang, Junhao Ding, Shuo Qu, Michael Yu Wang, Xu Song, Ming Wang Fu

**Affiliations:** ^1^ Department of Mechanical Engineering Hong Kong Polytechnic University Kowloon Hong Kong China; ^2^ Meta Robotics Institute and State Key Laboratory of Mechanical System and Vibration Shanghai Jiao Tong University Shanghai China; ^3^ Department of Mechanical and Automation Engineering Chinese University of Hong Kong Sha Tin Hong Kong China; ^4^ School of Engineering Great Bay University Songshan Lake Dongguan Guangdong China

**Keywords:** buckling‐resistant design, curvature directions, ribbed shell lattices, triply periodic minimal surfaces, ultralight and ultra‐strong lattices

## Abstract

Shell lattices are promising candidates for lightweight multifunctional applications for their geometric uniqueness and distinct mechanical properties. However, due to the transition from yielding‐to‐buckling failure mode, shell lattices exhibit notably reduced strength at low relative densities (RDs), making the design and optimization of ultralight and ultra‐strong shell lattices challenging. Herein, a novel design of rib‐reinforced shell lattices, namely ribbed shell lattices, is proposed to enhance the strength of ultralight triply periodic minimal surface (TPMS) shell lattices. By revealing the intrinsic relations between the curvature and stress directions of TPMS thin shell lattices, two groups of ribs are orchestrated along the representative curvature directions: the line of asymptotes (LOA) and the line of principal curvatures (LOC). Through physical realization and numerical simulations, incorporating ribs along the LOAs and LOCs that pass through the shell umbilical points is shown to enhance the strength by 112.3%, with an RD around 1.28%. Incorporating ribs can redistribute the stress and selectively strengthen thin shells to suppress their buckling deformation, especially at the umbilical region. The continuous ribs also provide additional load paths, resulting in improved load‐bearing efficiency. These findings provide a practical design method for rib‐reinforced ultralight and ultra‐strong shell lattices against micro‐architecture buckling failure.

## Graphical abstract

This study thoroughly reveals the relation between the curvature and stress direction of triply periodic minimal surface (TPMS) thin shell lattices and proposes a novel rib reinforcement design strategy to incorporate ribs along the line of asymptotes (LOA) and the line of principal curvatures (LOC) to enhance the strength of ultralight TPMS shell lattices by suppressing their micro‐architecture buckling deformation.

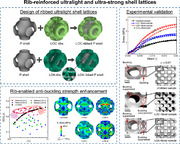

## 1 Introduction

The growing demand for lightweight structural applications has drawn considerable attention to lattice materials that are known for their low densities and superior mechanical properties [1, 2, 3, 4]. Triply periodic minimal surface (TPMS) shell lattices are a special class of lattice materials that can achieve a balance among open‐cell features, exceptional mechanical properties, and superior multifunctionality [5, 6, 7, 8]. However, lattice materials typically undergo a transition in failure mode from material yielding to micro‐architecture buckling as slenderness ratios increase and relative densities (RDs) decrease, resulting in a notable decrease in strength [9, 10, 11, 12]. Existing studies on TPMS shell lattices have mainly focused on enhancing their stiffness and yield strength, while the buckling‐strength‐driven design and optimization of ultralight shell lattices remain challenging due to their unique failure behavior.

Designing hierarchical micro‐architectures is an effective method to achieve superior buckling and compressive strength of ultralight lattices, since the hierarchical distribution of materials can enhance their effective bending stiffness and thus improve the buckling resistance [13]. The buckling strength of hierarchical lattices can be efficiently enhanced via a topology optimization framework to minimize the maximum of the first few most critical eigenvalues [14, 15] and through an integrated optimization workflow that concurrently considers global buckling instability and local buckling constraints [16]. However, these studies are typically based on the linear buckling analysis of hierarchical lattices, rather than enhancing their nonlinear compressive strength directly. In comparison, the compressive strength of hierarchical TPMS shell lattices can be directly improved by tuning the RDs and cell length ratios of their micro‐architectures to enhance the buckling stability, thus significantly outperforming their single‐scale counterparts at ultra‐low RDs [17]. The strength of hierarchical shell lattices can be further enhanced via the incorporation of conformal micro‐architectures [18] and the introduction of biometric bending‐ and stretching‐dominated micro‐architectures [19]. However, the additive manufacturability of hierarchical lattices tends to be significantly complicated by the different length scales of their micro‐architectures, which may differ by one to two orders of magnitude [17, 18].

In comparison, single‐scale shell structures generally possess superior additive manufacturability to their hierarchical counterparts and are gaining more attention [20, 21, 22]. In particular, the incorporation of ribs is an effective way to reinforce single‐scale shell structures. For instance, by incorporating ribs along the principal stress directions to enhance the load‐bearing efficiency, the stiffness and yield strength of thin‐walled shell structures can be significantly improved [23, 24]. To further enlarge the design space of rib‐reinforced shell structures, structural shape and topology optimization serves as a more generic design method, in which the ribs can be represented by a discrete geometric model [25, 26], a moving morphable component‐based framework [27], and a voxel density‐based method [28, 29]. These studies focused on the design of macroscopic shell structures under a single load case; however, shell lattices may be subjected to multiple load cases and their periodicity requires geometric smoothness between adjacent unit cells, both complicating the rib reinforcement design of shell lattices. Due to the lack of design guidelines, existing rib‐reinforced shell lattices are primarily based on rational design methods, such as the empirical [30] and bionic [31] design of ribbed Primitive (P)‐type TPMS shell lattices to achieve enhanced stiffness, strength, and crashworthiness. However, the optimal layouts and sizes of ribs, as well as the effects of ribs on the strength and failure behaviors of shell lattices, remain an open question.

In this study, we present our proposed novel rib reinforcement design for ultralight and ultra‐strong shell lattices, namely ribbed shell lattices. To find the optimal rib layouts, we revealed the intrinsic relations between the geometric curvature and stress directions of TPMS thin shell lattices through a novel B‐spline parameterized Monge patch geometric model and finite element analysis (FEA). The findings suggest generating ribs along the representative curvature directions of TPMSs: the line of asymptotes (LOA) and the line of principal curvatures (LOC). Through a systematic nonlinear FEA study on rib parameters, including the locations, thicknesses, and heights, we demonstrated that incorporating LOA and LOC ribs that pass through the umbilical region with maximum buckling deformation can enable the most significant strength enhancement of TPMS shell lattices. In particular, the strength of P‐type ribbed lattices can be enhanced by 70.2%∼112.3% at 1.18%∼1.28% RDs, and Neovius (N)‐type ribbed lattices can achieve 69.5%∼82.3% strength enhancement at 1.20%∼1.36% RDs. We demonstrated through numerical simulations that the incorporation of ribs can re‐distribute the stresses of lattices and selectively strengthen thin shells, especially at the umbilical region, which can effectively suppress their buckling deformation. Besides, the continuous ribs also provide additional load paths, thereby improving load‐bearing efficiency and enhancing the strength. Furthermore, a novel single‐track scanning strategy was utilized to fabricate the selected P‐type shell lattices by high‐resolution micro laser powder bed fusion (µLPBF), which enables the fabrication of ultralight metallic lattices with an RD around or lower than 2.0%, lower than those in most existing studies. Based on the compressive tests on high‐fidelity stainless steel 316 L (SS316L) samples fabricated, the LOA and LOC ribbed P‐type shell lattices were shown to exhibit 101.18% and 50.89% higher strength than that of the unribbed samples with an equal RD of 2.03%, respectively. Overall, these findings provide insights into the curvature and stress directions of TPMS thin shell lattices, offering a practical design method for buckling‐resistant ultralight and ultra‐strong shell lattices.

## 2 Results

### 2.1 Relations Between the Curvature and Stress Directions of TPMS Thin Shell Lattices

**FIGURE 1:**
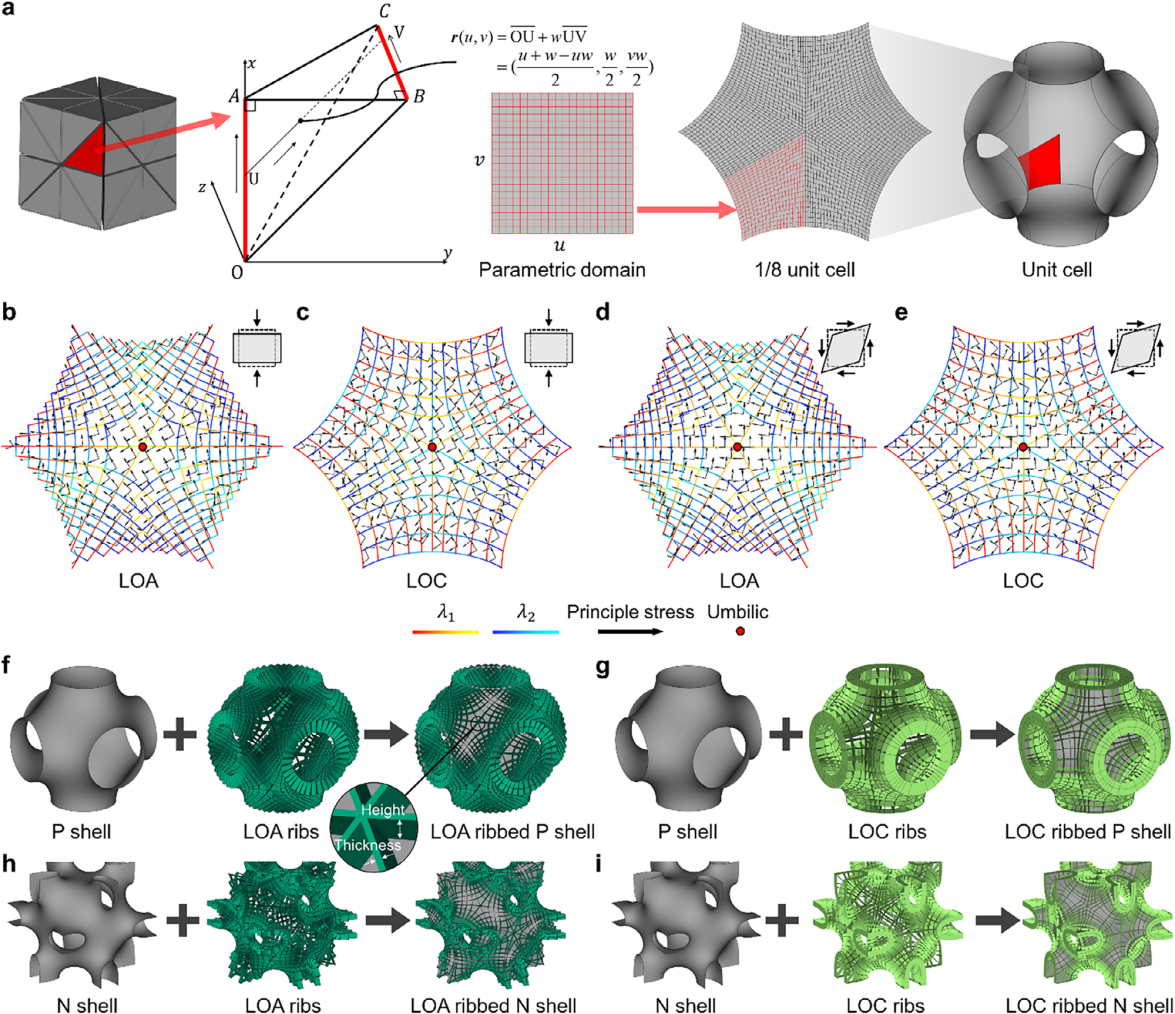
The geometric model and the intrinsic relations between the curvature and stress directions of TPMS thin shell lattices. (a) The B‐spline parameterized Monge patch model of P‐type TPMS and the correlations between the principal stress directions and the (b) LOA and (c) LOC directions under macroscopic uniaxial strain loading, and (d, e) pure shear loading. Representative designs of P‐type (f) LOA and (g) LOC ribbed shell lattices, and N‐type (h) LOA and (i) LOC ribbed shell lattices.

To determine the optimal rib locations, it is essential to find the intrinsic relationship between the geometric curvature and the mechanical responses of shell lattices. Here, we correlated the representative curvature directions, i.e., LOA and LOC, with the principal stress directions according to the membrane theory of thin shells. To accurately compute the geometric curvatures of TPMSs, a versatile B‐spline parameterized Monge patch model was developed (Figure 1a). Utilizing the cubic symmetry of lattices, the unit cell was simplified to a fundamental tetrahedral domain (1/48‐unit cell). For the P‐type TPMS, the basic patch was parameterized as in Figure 1a. By solving a second‐order partial differential equation in its weak form that pursues a constant zero mean curvature, namely ∇_s_ · ***n***  =  0, where ∇_s_ is the surface gradient operator and ***n*** is the surface normal, highly accurate minimal surfaces can be obtained (elaborated in the “Methods” section). The Monge patch model enables highly accurate and efficient computation of their geometric characteristics, such as the LOA and LOC (Figure 1b–e; Figure S1b). The principal stress directions of P‐type TPMS shell lattices with an RD of 2.5% under two representative load cases, namely macroscopic uniaxial strain (Figure 1b,c) and pure shear (Figure 1d–e) loadings, are overlayed and compared with the LOA and LOC directions. The comparison shows that the principal stress directions mostly align with the LOA directions, regardless of load cases. The quantitative angular deviation computations show that the angular deviations of 73.5% and 85.7% elements are lower than 15° under uniaxial strain and pure shear stress states, respectively (Figure S2d). The remaining deviations are due to the bending effects of shells and the indeterminacy of the principal curvature directions at the umbilical point, where the curvatures are equal (being zero) in all directions. These findings are confirmed by the membrane theory of thin shells for the special case of TPMS shell lattices [32], indicating that the normal stresses in LOA coordinates are along the principal stress directions, with vanishing shear stresses. In addition, the LOC directions differ from the LOA directions by an angle of 45°, implying a state of maximum shear stress (with equal normal stresses) in LOC coordinates (Figure S2). The intrinsic relationship between the geometric curvature and the principal stress directions provides theoretical guidelines for designing ribs aligned with the LOA and LOC directions.

Based on the computed curvature directions together with the normal vector, ribs were orchestrated along the LOA and LOC directions of TPMS shell lattices. The LOA and LOC are representative features of surfaces, in which the normal curvature vanishes along the LOA direction, and the torsion vanishes along the LOC direction. For minimal surfaces, in particular, the two groups of LOAs (denoted as *λ*_1_ and *λ*_2_) are perpendicular to each other except for the umbilics (Figure 1b,c), and so are the two groups of LOCs (Figure 1d,e, denoted as *λ*_1_ and *λ*_2_). The unit cells of P‐type TPMSs with LOA and LOC ribs are depicted in Figure 1f,g and Figure S3a,b, showing that the ribs are smooth within the unit cell and the cell boundaries due to the high accuracy of the geometric model. To demonstrate the versatility of the geometric model, the N‐type TPMSs, as well as the corresponding LOA and LOC ribs, are also orchestrated and shown in Figure 1h,i and Figure S3c,d. The proposed geometric model can be straight forwardly extended to other TPMSs according to their space groups and the corresponding fundamental domains [33, 34].

### 2.2 Rib‐Reinforced Ultralight Shell Lattices by Tuning Micro‐Architecture Buckling Deformation

**FIGURE 2:**
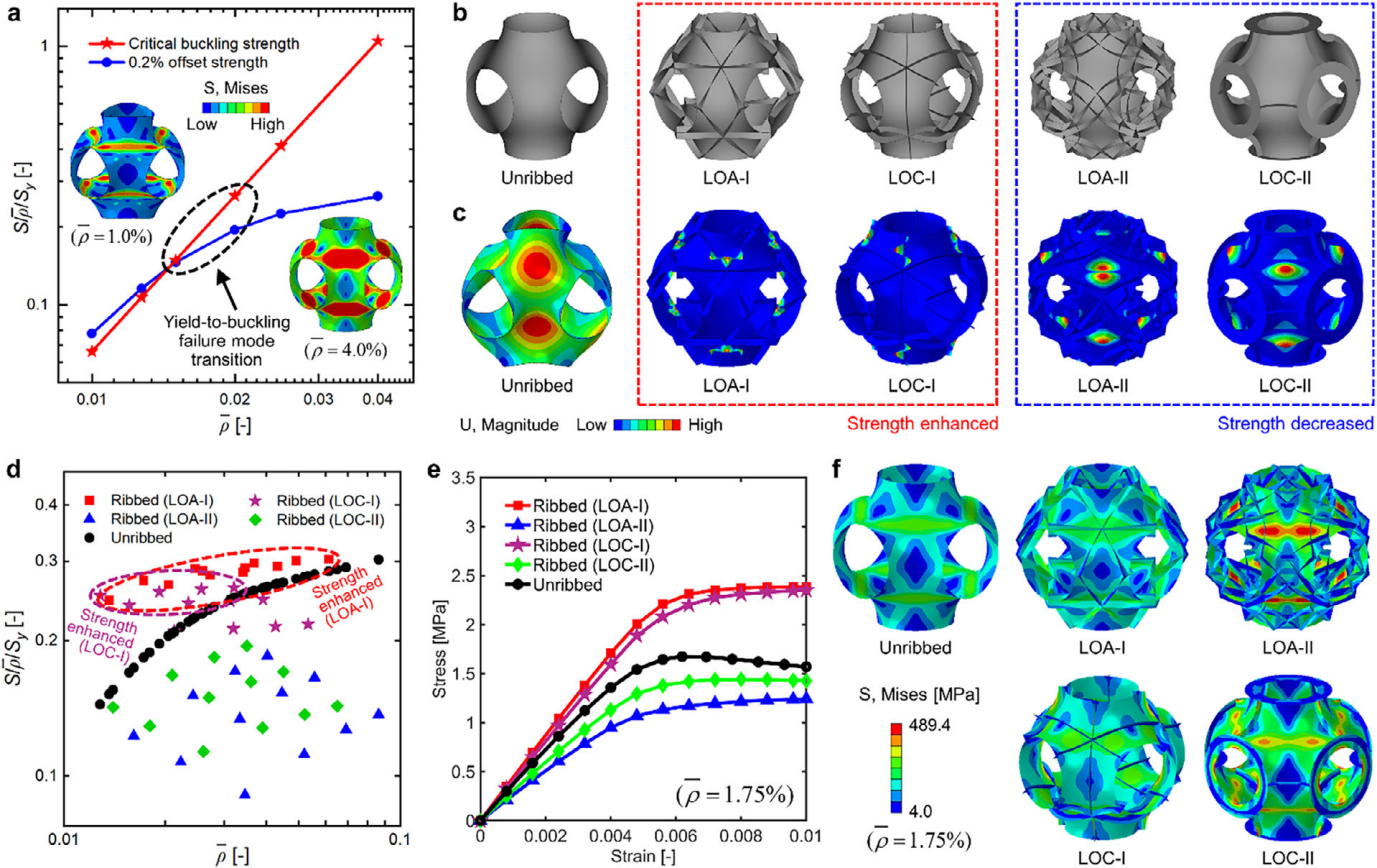
Rib‐reinforced design of ultralight P‐type TPMS shell lattices. (a) The yielding‐to‐buckling failure mode transition of P‐type shell lattices, (b) the unribbed P‐type shell lattices and two representative layouts of LOA and LOC ribbed lattices, and (c) their critical buckling modes. (d) The normalized strength (S/$\bar{\rho }$/S_y_) versus RD ($\bar{\rho }$) plots of unribbed, LOA ribbed, and LOC ribbed lattices, and their (e) stress‐strain curves and (f) von Mises stress contours (under a macro stress of 1.0 MPa) with an RD of 1.75%.

The P‐type TPMS shell lattices undergo a failure mode transition from material yielding to micro‐architecture buckling in the 1.5%∼2.0% RD range. The transition is manifested as a sudden change in the slope of the normalized strength (*S*/$\bar{\rho }$/*S_y_*), which is defined as the ratio of the strength (*S*) of the lattices to the Voigt strength upper bound of anisotropic cellular materials ($\bar{\rho }S_{y}$) [6], versus RD ($\bar{\rho }$) plot in logarithmic coordinates, and is further validated by a significant difference in the deformation state and stress distribution of the lattices with different RDs (Figure 2a; Figure S4). Specifically, the edge of the lattice with 1.0% RD undergoes distortional deformations, and the region near the umbilical point bulges out or is dented significantly, matching well with the critical buckling mode and implying a micro‐architecture buckling failure mode. In contrast, no significant distortions occur in the edge of the lattice with 4.0% RD, and the umbilical region exhibits a high level of stress that exceeds the yield strength of the constitutive material, indicating a material yielding failure mode (Figure 2a). Due to the yielding‐to‐buckling failure mode transition, the buckling strength‐driven design method should be developed for ultralight shell lattices.

The LOA and LOC ribs were orchestrated and incorporated onto P‐type TPMS shell lattices to enhance the strength (Figure S3a,b). Two representative layouts of LOA ribbed lattices, namely LOA‐I and LOA‐II, are elaborated herein, representing the cases in which incorporating ribs enhances and decreases the strength of shell lattices, respectively; and so do the two layouts of LOC‐I and LOC‐II ribbed lattices (Figure 2b). The incorporation of ribs remarkably affects the buckling deformation, in which the critical buckling modes of ribbed lattices can be categorized into two classes. Specifically, the LOA‐I and LOC‐I ribs pass directly through the umbilical point with maximum buckling deformation, thus suppressing the shell buckling deformation efficiently; and the buckling deformation of the ribbed lattices mainly occurs in the ribs (Figure 2c). In contrast, the incorporation of LOA‐II and LOC‐II ribs that are located away from the umbilical region cannot effectively suppress the buckling deformation of shell lattices, and the buckling deformation still occurs in the umbilical region of the shell primarily (Figure 2c). Therefore, the LOA‐I and LOC‐I ribbed lattices can achieve superior strength enhancement over the LOA‐II and LOC‐II layouts.

The effects of rib layouts on the compressive strength of P‐type shell lattices are further elaborated for the representative design of ribbed lattices (Figure 2d–f; Figures S5 and S6). The strength can be enhanced by incorporating LOA‐I and LOC‐I ribs, and the magnitude of enhancement shows an overall increasing trend with the decrease of RD. In particular, the LOA‐I ribbed lattice achieves 62.0% strength enhancement at 1.36% RD, and the LOC‐I ribbed lattice gets 112.3% enhancement at 1.28% RD. In contrast, the LOA‐II and LOC‐II ribbed lattices exhibit lower strength than their unribbed counterparts with equal RDs (Figure 2d,e). In fact, the incorporation of ribs can re‐distribute the stresses, which further explains the reason for the strength enhancement or decrease of the lattices (Figure 2f). Specifically, the maximum local stress of the shell of LOA‐I and LOC‐I ribbed lattices is effectively reduced. The high stress state of the rib indicates that the rib also bears loads efficiently, thus resulting in enhanced strength (Figure 2f). In comparison, the weakening effect of LOA‐II and LOC‐II ribbed lattices is demonstrated by the low stress magnitudes of the ribs and a remarkable increase in the maximum local stress of the shell as compared to the unribbed lattice. Thus, the two rib layouts cannot suppress the shell buckling deformation effectively, leading to decreased load‐bearing efficiency and lower strength (Figure 2f). Moreover, the LOC‐I and LOC‐II ribs can be regarded as two intuitive rib layouts, especially the LOC‐I, which serves as a more straightforward configuration of cross‐shaped ribs aligned with the loading direction. As a comparison, the LOA‐I ribbed lattices can achieve more exceptional strength than that of LOC‐I ribbed lattices with equal RDs (Figure 2d), since the LOA‐I rib is approximately aligned with the principal stress direction, and enables more effective buckling suppression at ultra‐low RDs (Figure 2f). In this study, among all of the rib groups shown in Figure 1f–i, the effect of each individual group of ribs is studied to identify the rib group that contributes most to the strength enhancement, respectively. Overall, ribs passing through the umbilical points of TPMSs, which is also the region with maximum buckling deformation, can effectively tune the shell buckling deformation and hence enhance the compressive strength of ultralight shell lattices. While the spacing of ribs within a specific group does not show a clear correlation to the strength enhancement, it is interesting to incorporate multiple rib groups into ultralight shell lattices, and the spacing between different rib groups could be an important design variable that affects the strength enhancement. The coupling effects of different rib groups on the strength of ultralight shell lattices remain an open question and will be studied in detail in our future works.

### 2.3 Optimization of Rib Height and Thickness for Strength Maximization

**FIGURE 3:**
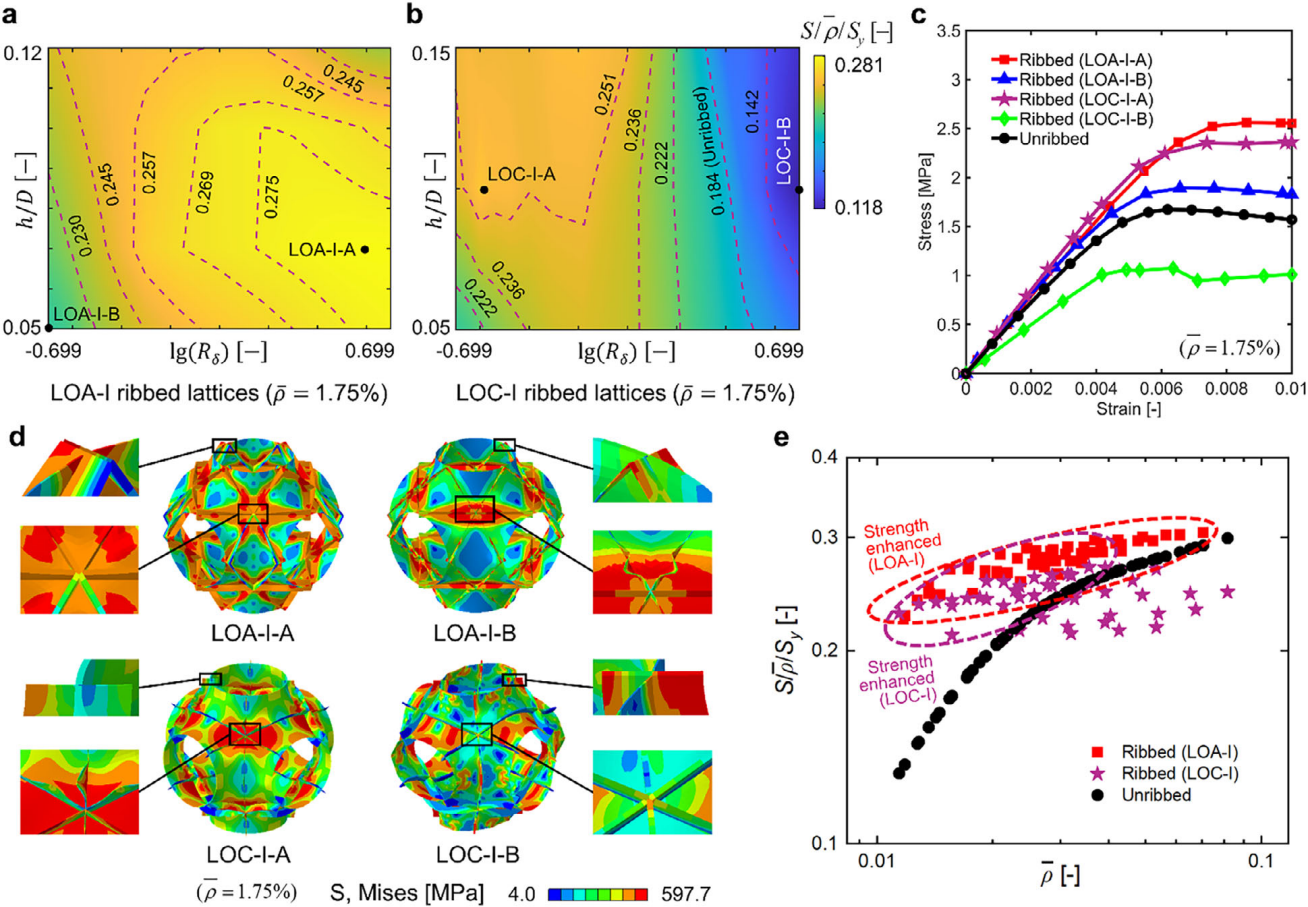
Optimization of the rib height and thickness to maximize the strength of P‐type ribbed TPMS shell lattices. The normalized strength of (a) LOA‐I and (b) LOC‐I ribbed P‐type shell lattices with varying rib heights and thicknesses, (c) the stress‐strain curves, and (d) von Mises stress contours (under a macro compressive strain of 0.01) of four representative layouts of ribbed lattices with an equal RD of 1.75%. (e) The normalized strength versus RD plots of LOA‐I and LOC‐I ribbed lattices with varying rib heights and thicknesses, and those of their unribbed counterparts with equal RDs.

The height and thickness of ribs were further optimized to maximize the strength of LOA‐I and LOC‐I ribbed P‐type shell lattices (Figure 3a–d; Figures S5 and S6). Typically, the strength of lattice materials depends on the interplay between geometric and material nonlinearities, and finding the global optimum is difficult. In this case, by considering the manufacturability constraints of ribbed shell lattices, especially the rib‐to‐shell feature size ratio, a sufficiently large parameter space for the rib height and thickness was selected (Figure 3a,b). Given an RD of 1.75%, the LOA ribbed lattices can all achieve enhanced strength than their unribbed counterparts within the considered structural parameter range (Figure 3a). The maximum strength enhancement of LOA ribbed lattices occurs when the rib to shell thickness ratio (*R*_*δ*_) is nearly 4.0, and the rib height to unit cell size ratio (*h*/*D*) is 0.07 (LOA‐I‐A). Under a macro compressive strain of 0.01, the shell buckling deformation of LOA‐I‐A ribbed lattices is effectively suppressed, and the rib is in a high stress state to bear loads efficiently (Figure 3d), thereby resulting in the highest magnitude of strength enhancement. However, as the rib thickness decreases, the rib gradually undergoes significant buckling deformations, leading to a lower magnitude of strength enhancement (LOA‐I‐B, Figure 3c–d). In particular, the LOA‐I ribbed lattices can achieve 52.55% strength enhancement in maximum at 1.75% RD, achieving a greater magnitude of enhancement than that in Figure 2d.

The optimization of rib height and thickness can also further enhance the strength of LOC ribbed lattices. Specifically, the maximum strength enhancement of LOC‐I ribbed lattices occurs when *R*_*δ*_ is nearly 0.25, and *h*/*D* is 0.10 (LOC‐I‐A, Figure 3b). The LOC‐I‐A ribbed lattices, although undergoing evident buckling deformation in the rib, can achieve remarkably enhanced strength via tuning the shell buckling deformation, manifested as no significant deformity in the shell edge region (Figure 3d). However, the LOC‐I‐B ribbed lattices, despite of possessing a larger rib thickness and exhibiting no evident buckling deformation in the rib, cannot effectively suppress the shell buckling deformation (as illustrated by the distorted shell edge region), thus leading to a significant decrease in strength (Figure 3c,d). In particular, the LOC‐I ribbed lattices can achieve up to 40.77% strength enhancement at 1.75% RD, higher than that in Figure 2d. Overall, the strength enhancement magnitude of both LOA and LOC ribbed lattices can be further improved by optimizing the rib height and thickness, resulting in a local optimum that is manufacturable and can achieve significantly enhanced strength than their unribbed counterparts.

The normalized strengths of LOA‐I and LOC‐I ribbed P‐type shell lattices with varying structural parameters (including rib heights and thicknesses) are further compared to those of the unribbed lattices with equal RDs (Figure 3e). Generally, the LOA and LOC ribbed lattices significantly outperform their unribbed counterparts in strength, especially in the ultra‐low RD regime. In particular, the LOA ribbed lattices can achieve 11.97%∼52.55% strength enhancement at 1.75%∼4.23% RDs; the LOC ribbed lattices can achieve a maximum of 40.77% strength enhancement at 1.75% RD, and exhibit a minimum of 14.32% lower strength at 3.20% RD. Overall, the comparison further demonstrates the superior strength of the rib‐reinforced ultralight shell lattices to their unribbed counterparts.

### 2.4 Experimental Validation of the Rib Reinforcement Effect

**FIGURE 4:**
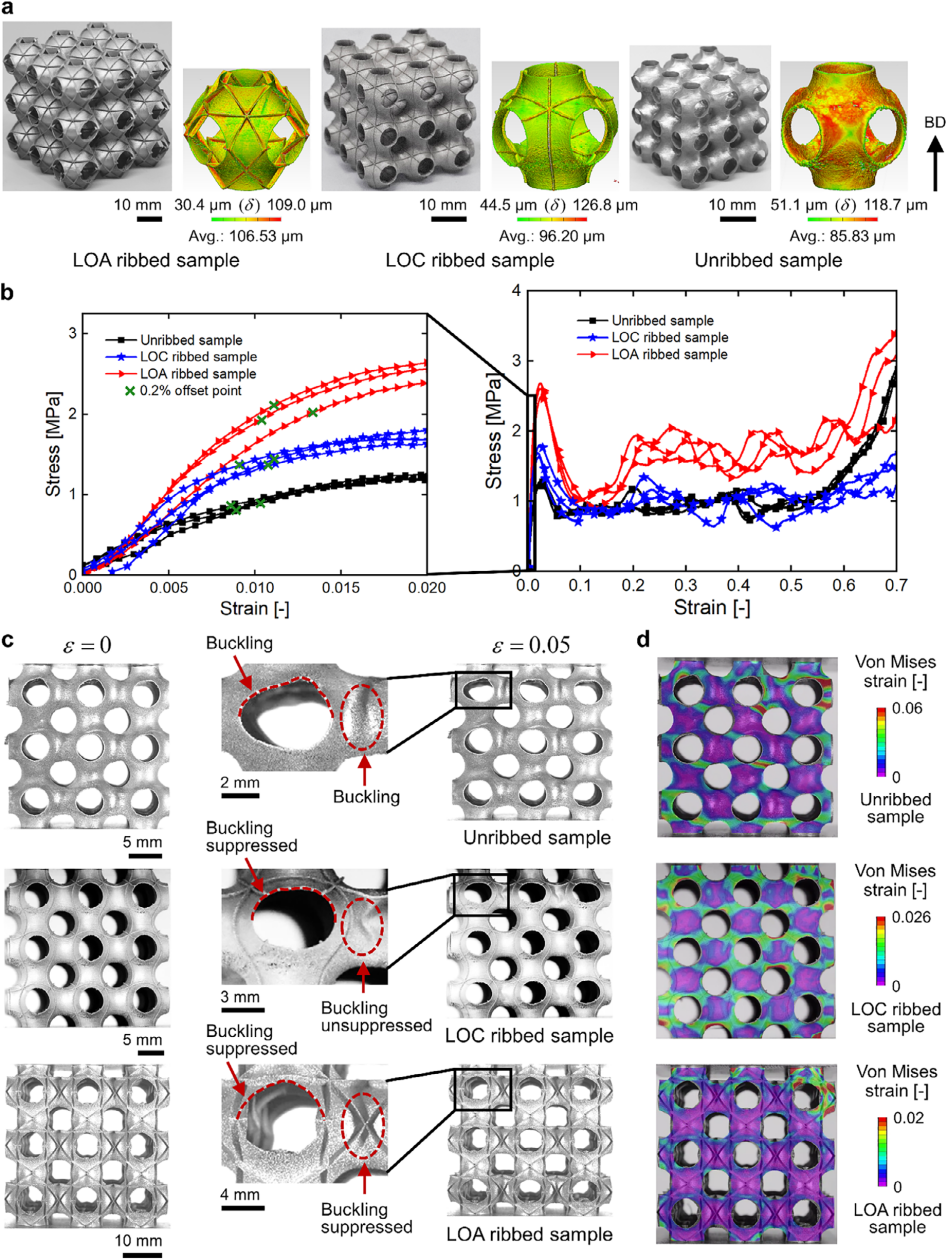
Compression test results of the high‐fidelity SS316L samples of LOA ribbed, LOC ribbed, and unribbed P‐type TPMS shell lattices. (a) The high‐fidelity samples of LOA ribbed, LOC ribbed, and the unribbed P‐type shell lattices and the micro‐CT characterization of their wall thickness (*δ*) distributions, in which the arrow denotes the building direction (BD), the experimentally measured (b) stress‐strain curves and (c) deformation patterns under 0 and 0.05 compressive strains, and (d) the von Mises strain distributions evaluated through the DIC analysis.

In this study, the P‐type TPMS shell lattices were selected for manufacturing further to validate the LOA/LOC rib reinforcement design strategy. In contrast, the N‐type shell lattices encounter challenges in the printing efficiency and are mainly explored via numerical simulations, since the N‐type TPMS possesses a much larger specific surface area and requires additional rotational operations to overcome the overhang constraints. The unribbed, LOA ribbed, and LOC ribbed P‐type shell lattices with an RD of 2.03% were fabricated via high‐resolution µLPBF processes [35, 36] in SS316L. Based on the high‐fidelity metallic samples fabricated (Figure 4a), the mechanical properties of the lattices were measured by quasi‐static compression tests, as illustrated in Figure 4b,c. The experimentally measured average RDs of LOA ribbed, LOC ribbed, and the unribbed samples are 2.86%± 0.03%, 2.58%± 0.03%, and 2.39%± 0.04%, respectively, which are higher than the as‐designed RD (2.03%). The discrepancy is primarily attributed to the larger wall thicknesses of the fabricated samples than those of the designed geometries, and the thickness deviations of ribs are generally greater than those of shells. The micro‐CT characterized wall thickness (*δ*) distributions of the as‐fabricated samples demonstrate that the average thicknesses of the LOA ribbed, LOC ribbed, and unribbed samples are 106.53, 96.20, and 85.83 µm, respectively (Figure 4a). Compared to the as‐designed average thicknesses of 75.39, 75.57, and 70 µm, the thickness deviations between the as‐fabricated samples and the as‐designed geometries are within an acceptable range (Section S12, Supporting Information).

The experimental stress‐strain curves reveal that incorporating LOA and LOC ribs can both significantly enhance the strength of P‐type TPMS shell lattices. The experimentally measured normalized strengths (*S*/$\bar{\rho }$/*S_y_*) of the LOA and LOC ribbed samples are 0.136± 0.006 and 0.104± 0.003, which are 94.29% and 48.57% higher than that of the unribbed samples (0.070± 0.003), respectively. The LOA ribbed samples exhibit a higher magnitude of strength enhancement than that of the LOC ribbed samples (Figure 4b), matching well with the numerical results. The LOA and LOC ribs are able to selectively enhance the local bending stiffness and strength of thin shell lattices, especially at the low‐curvature and free edge regions, which can effectively suppress the shell buckling deformation and enhance the buckling and compressive strength of thin shell lattices. Specifically, the unribbed shell lattices experienced buckling deformation under a compressive strain of 0.05, characterized by significant distortional deformations at the free edge regions and the dents at the cell connection regions (Figure 4c). In comparison, the LOC ribs pass through the edges and can efficiently tune the deformations from distortion to bending. However, the connecting regions between adjacent unit cells remained dented, implying that the LOC ribs cannot effectively suppress the shell buckling deformation at these regions (Figure 4c). As for the LOA ribbed samples, no significant distortions were observed at the free edge regions under the same compressive strain, and the connecting regions between adjacent unit cells were no longer dented. Therefore, the incorporation of LOA ribs was shown to be capable of suppressing the shell buckling deformation in more regions, thereby achieving higher strength enhancement than that of the LOC ribbed samples (Figure 4c). Moreover, the experimentally observed post‐buckling deformation patterns (Figure 4c) exhibit clear consistency to the numerically predicted critical buckling modes (Figure 2c) of the lattices, which further demonstrates the rib‐enabled strength enhancement of ultralight shell lattices via buckling suppression.

In addition, the digital image correlation (DIC) analysis reveals that the unribbed samples exhibit prominent local buckling deformations at the low curvature umbilic regions, as illustrated by the red and green regions in Figure 4d, which leads to distortions at the free edge regions, agreeing with the numerical simulations reasonably. When the LOC ribs are introduced, the localized deformations at the umbilic regions are defused. The deformation magnitude is significantly reduced, as shown in the wide range of green regions in Figure 4d. For the LOA ribbed samples, the local buckling deformations at the umbilic and free edge regions are further suppressed and the von Mises strain distributions become more uniform (Figure 4d). Therefore, the DIC analysis results further validate that the suppression of local buckling deformation by LOA and LOC ribs results in significant strength enhancement of ultralight shell lattices. Additionally, the normalized plateau stresses (NPSs, *σ*_*p*_) and specific energy absorption (SEA, *ψ*) of the samples were evaluated based on the experimental stress‐strain curves. The LOA ribbed samples exhibit 46.84% and 83.45% higher NPS and SEA than the unribbed samples, while the NPS and SEA of LOC ribbed samples do not change too much as compared to the unribbed samples (Figure S15). The mechanical properties of the three repetitive samples within each group exhibit a low magnitude of relative standard deviation, validating the repeatability of experimental data based on the µLPBF‐fabricated high‐fidelity samples. Overall, the experimental results demonstrated that the strength of ultralight P‐type shell lattices can be significantly improved by incorporating LOA and LOC ribs to suppress the shell buckling deformation, validating the proposed design guideline of rib‐reinforced buckling‐resistant ultralight and ultra‐strong shell lattices.

### 2.5 Extension to Other Types of TPMS Shell Lattices

**FIGURE 5:**
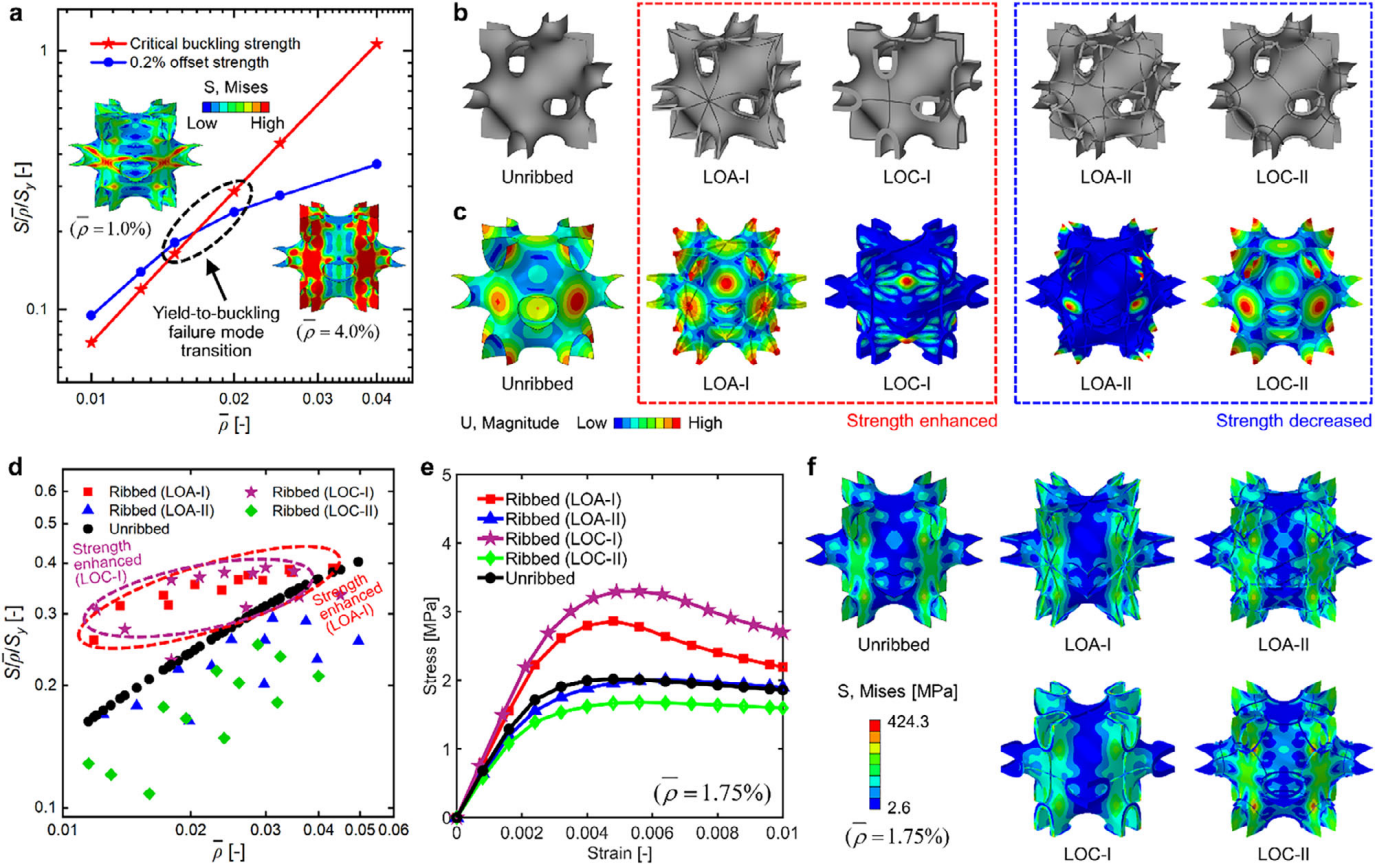
Rib‐reinforced design of ultralight N‐type TPMS shell lattices. (a) The yielding‐to‐buckling failure mode transition of N‐type shell lattices, (b) the unribbed N‐type shell lattices and two representative layouts of LOA and LOC ribbed lattices, and (c) their critical buckling modes. (d) The normalized strength versus RD plots of unribbed, LOA ribbed, and LOC ribbed lattices, and their (e) stress‐strain curves and (f) von Mises stress contours (under a macro stress of 1.0 MPa) with an RD of 1.75%.

The rib‐reinforced design of ultralight N‐type TPMS shell lattices is further explored to illustrate the method's generality. Through nonlinear FEA simulations, N‐type shell lattices are shown to undergo the yielding‐to‐buckling failure mode transition in the 1.5%∼2.0% RD range (Figure 5a and Figure S11). Accordingly, the LOA and LOC ribs were incorporated to seek the enhancement in strength (Figure S3c∼d), in which four representative layouts are elaborated in Figure 5b, and the other layouts are illustrated in Figure S12. The N‐type TPMSs are found to possess more umbilical points than those of P‐type TPMSs, and the critical buckling mode of unribbed N‐type shell lattices reveals that the umbilical regions, which are also flat, undergo severe out‐of‐plane deformations. Accordingly, incorporating LOA‐I and LOC‐I ribs that pass directly through the umbilical points can efficiently tune the shell buckling deformation by suppressing their out‐of‐plane deformations. However, the shell buckling deformation cannot be effectively suppressed by LOA‐II and LOC‐II ribs, which are located away from the umbilical regions (Figure 5c). Therefore, the LOA‐I and LOC‐I ribbed lattices can achieve enhanced strength compared to their unribbed counterparts, especially at lower RDs, while incorporating LOA‐II and LOC‐II ribs tends to decrease the strength (Figure 5d–e). Specifically, incorporating LOA‐I and LOC‐I ribs can effectively reduce the maximum local stress in the shell, and the ribs also bear loads efficiently, resulting in improved load‐bearing efficiency. However, the incorporation of LOA‐II and LOC‐II ribs has an opposite effect and leads to decreased strength (Figure 5f). In particular, N‐type ribbed lattices can achieve up to 69.5%∼82.3% strength enhancement at 1.20%∼1.36% RDs. Overall, the results of N‐type ribbed shell lattices validate again the proposed rib reinforcement design method of buckling‐resistant ultralight and ultra‐strong shell lattices.

## 3 Discussion

To summarize, we proposed a novel rib reinforcement design of ultralight shell lattices, namely ribbed shell lattices, to locally strengthen thin shells and enhance their buckling and compressive strength. Two representative groups of ribs, namely LOA and LOC ribs, were orchestrated based on the intrinsic relations between the curvature and stress directions of TPMS thin shell lattices. Based on the membrane theory of thin shells, the principal stress direction is found to be aligned with the LOA, while a state of maximum shear stress (with equal normal stresses) is observed along the LOC, regardless of load cases. By incorporating LOA and LOC ribs that pass through the umbilical points, the strength of TPMS shell lattices can be significantly enhanced. The incorporation of LOA ribs generally poses a higher chance of resulting in greater strength enhancement than that of LOC ribs. The strength enhancement arises from the capability of incorporating appropriate rib layouts to re‐distribute the stresses of lattices and selectively strengthen thin shells to suppress their buckling deformation, especially at the umbilical region. The continuous ribs also provide additional load paths, thereby improving the load‐bearing efficiency and enhancing the strength. The magnitude of strength enhancement can be further improved by optimizing the rib thickness and height. In particular, P‐type ribbed lattices can achieve 70.2%∼112.3% strength enhancement at 1.18%∼1.28% RDs, and the strength of N‐type ribbed lattices can be enhanced by 69.5%∼82.3% at 1.20%∼1.36% RDs. The compression tests on high‐fidelity SS316L samples of P‐type shell lattices further demonstrated that the LOA and LOC ribbed samples outperform their unribbed counterparts in strength by 101.18% and 50.89%, respectively. Overall, these findings offer an effective design method for buckling‐resistant ultralight and ultra‐strong shell lattices, and can serve as a design guideline for ultralight engineering materials with enhanced strength. Compared to hierarchical TPMS shell lattices [17], the proposed rib‐reinforced ultralight shell lattices share the same underlying principle of buckling suppression for strength enhancement. Typically, the rib‐reinforced designs exhibit superior additive manufacturability to their hierarchical counterparts [17, 18], and can achieve a greater degree of strength enhancement due to the more effective buckling suppression effect (Figure S13). Moreover, the membrane thin shell theory is demonstrated to hold in most regions of TPMS thin shell lattices, regardless of load cases, making the LOA/LOC rib‐reinforcement method a generic design strategy across different load cases.

The rib‐reinforced ultralight and ultra‐strong shell lattices demonstrate broad application prospects across aerospace, automotive, thermal control, and healthcare. In particular, these lattices satisfy the requirements for lightweight aerospace components, significantly reducing raw material costs and enhancing their strength and load‐bearing capacity. The incorporation of appropriate rib layouts can also enhance the fracture toughness and fatigue life of ultralight shell lattices by inhibiting fatigue crack propagation under repetitive or cyclic loading, thereby improving their long‐term service capability [37, 38]. This aspect necessitates the establishment of a multiscale analysis framework to evaluate the fracture and fatigue behaviors of lattice materials and to reveal the effects of rib layouts on the fracture toughness and fatigue life of ribbed shell lattices. Moreover, the application of rib‐reinforced ultralight shell lattices necessitates scalable additive manufacturing, in which a reasonable balance must be struck between the fabrication quality and efficiency, especially for the mass production of large‐scale engineering structures. In this case, the stereolithography (STL)‐free additive manufacturing procedure can play a significant role, in which the implicit solid modelling and direct slicing processes can be seamlessly integrated, thereby avoiding the STL mesh‐related intermediate steps [39]. The STL‐free workflow can save the memory and storage related to STL files for over 90% and ensure the fabrication quality simultaneously. The fabrication quality can be further improved by integrating the STL‐free workflow with an optimized laser scanning strategy, with the energy deposition trajectories precisely regulated to enhance the surface quality of ultralight shell lattices and enable the satisfaction of manufacturing tolerance limits [40]. These aspects will be studied in detail in our upcoming works.

Thus far, existing studies on TPMS shell lattices have mainly focused on the design to enhance their stiffness and yield strength, which typically follows the conventional ‘stretching‐domination’ design principle [41, 42]. However, lattice materials tend to undergo the yielding‐to‐buckling failure mode transition with increasing slenderness ratios and decreasing RDs [9, 10, 11, 12], while the buckling strength‐driven design and optimization of ultralight shell lattices remain less explored. In this study, we revealed the critical role of enhancing the buckling stability for improving the strength of ultralight shell lattices through the proposed rib reinforcement design strategy. Although this study focuses on the relatively symmetric P‐ and N‐type TPMSs, the design strategy can be readily extended to other families of surfaces with the Monge patch model representation. In contrast, the Monge patch models of less symmetric geometries, such as Gyroid‐family surfaces, are not readily available. In this case, the implicit periodic nodal surface approximation can be utilized for the LOA/LOC rib reinforcement design strategy, in which the higher‐order terms should be incorporated to ensure the geometric accuracy. The rib reinforcement design strategy can also be applied for other constitutive materials (Figure S10), such as ceramic [3, 43] and pyrolytic carbon [44], which possess higher strength to Young's modulus ratios (*S_y_*/*E_s_*). Typically, these brittle lattices undergo the failure mode transition from material fracture to micro‐architecture buckling at larger RDs, leading to a more pronounced strength reduction than that of metallic lattices. For example, SiOC ceramic lattices (*S_y_*/*E_s_* ≈ 0.1) tend to undergo the failure mode transition at an RD near 27.0% [3, 43], and pyrolytic carbon plate lattices (*S_y_*/*E_s_* ≈ 0.04) may undergo the transition with the RD around 37.5% [44]. In this case, the rib reinforcement design strategy can enable more effective buckling suppression and strength enhancement within a wider range of RDs. However, the rib reinforcement effect no longer holds at moderate RDs (Figures 2d and 5d), in which material yielding takes over as the dominant failure mode of the lattices, and the strengthening strategy should in turn follow the conventional ‘stretching‐domination’ design principle. Accordingly, based on the curvature‐stress relation revealed in this study, the curvature and local stress of shell lattices can be re‐distributed via structural shape and topology optimization approaches to achieve a more stretching‐dominated deformation state for strength enhancement. These studies will be thoroughly conducted in our future endeavors to enlarge the design space of shell lattices to further enhance their strength.

## 4 Methods

### 4.1 Modeling Method: B‐spline Parameterized Monge Patch Model

The proposed geometric model of TPMSs was developed on an irreducible fundamental domain. The fundamental domains of P‐ and N‐type TPMSs are identical: the quadrirectangular tetrahedron, i.e., the 1/48‐unit cell (Figure S1a). Subsequently, the unit cell of TPMSs was created from the fundamental domain via mirror operations [33]. A 2D parametric domain (*u*, *v*)∈[0, 1]^2^ was defined for the surface according to its topology. The surface model was referred to as a Monge patch model, as the parametrization defines the surface by its height on a flat 2D parametric domain. To guarantee the smoothness and differentiability, an implicit B‐spline surface model was established. The B‐spline parameterized Monge patch model of P‐family surfaces is defined as [33]:

(1) $$\begin{array}{ccc} r=r\;\left(u,v,w\right) & = & \bar{\mathrm{OU}}+w\;\bar{\mathrm{UV}}\\ & = & \left(\frac{u+w-\mathrm{uw}}{2},\frac{w}{2},\frac{\mathrm{vw}}{2}\right),\left(u,v\right)\in {\left[0,1\right]}^{2}, \end{array}$$

in which *w* is a B‐spline function of the other two parameters *u* and *v*:

(2) $$w=w\;\left(u,v\right)=\sum_{i=1}^{n}\sum_{j=1}^{m}N_{i,p}\left(u\right)M_{j,q}\left(v\right)C_{\mathrm{ij}},\left(u,v\right)\in {\left[0,1\right]}^{2},$$

where *C_ij_* are unknown control coefficients, and *n* and *m* are the numbers of control coefficients along *u* and *v* directions, respectively. Besides, *N_i,p_* (*u*) and *M_j,q_* (*v*) are the B‐spline basis functions, and *p* and *q* are the polynomial degrees (*p* = *q* = 3 was used in this study). For N‐family surfaces, the Monge patch model in Equation (1) should be replaced by [34]:

(3) $$\begin{array}{ccc} r=r\left(u,v,w\right) & = & \bar{\mathrm{OU}}+w\bar{\mathrm{UV}}\\ & = & \;\left(\frac{u+w\;-\;\mathrm{uw}}{2},\frac{u\;-\;\mathrm{uw}+\mathrm{vw}}{2},\frac{\mathrm{vw}}{2}\;\right),\left(u,v\right)\in {\left[0,1\right]}^{2}. \end{array}$$

To obtain a minimal surface, the second‐order partial differential equation (PDE) ∇_s_ · ***n*** + 2*H*  =  0 was solved to determine the unknown control coefficients, where *H* is the prescribed mean curvature (being zero). Using the Galerkin method, the weak form of the governing equation is stated as [34]:

(4) $$\int \int_{S}\phi_{\mathrm{ij}}\left(\nabla_{s}\cdot n+2H\right)\mathrm{dA}=0,\left(i=1,..n;\;j=1,..,m\right),$$

where *ϕ*_*ij*_ = *N*_*i*,*p*_ (*u*)*M*_*j*,*q*_(*v*), and ∇_s_ is the surface gradient operator. The second‐order PDE can be converted to a first‐order PDE using the surface divergence theorem [34]:

(5) $$\begin{array}{ccc} & & \int \int_{S}\nabla_{s}\cdot F_{\mathrm{ij}}\mathrm{dA}\\ & & \;=\int_{\partial S}m\cdot F_{\mathrm{ij}}\;\mathrm{ds}-\int \int_{S}2Hn\cdot F_{\mathrm{ij}}\mathrm{dA},\left(i=1,..n;\;j=1,..,m\right), \end{array}$$

where the test function is chosen as $F_{\mathrm{ij}}=\frac{\partial r}{\partial w}\;\phi_{\mathrm{ij}}$, ***m*** = ***t*** × ***n*** with ***t*** being the unit tangent vector of the boundary curve, and ***n*** = $\frac{r_{u}\times r_{v}}{\left|\right|r_{u}\times r_{v}\left|\right|}$ is the unit normal vector. According to the periodicity and symmetry of TPMSs, the surface should be orthogonal to the mirror planes at the boundary ∂*S*, i.e., ***m*** ·  ***F**_ij_* =  0. Therefore, the governing equation in Equation (5) can be solved by a minimization problem of the residuals *R_ij_* [34]:

(6) $$R_{\mathrm{ij}}=\int \int_{S}\left(\nabla_{s}\cdot F_{\mathrm{ij}}+2Hn\cdot F_{\mathrm{ij}}\right)\mathrm{dA}\;,\left(i=1,..n;\;j=1,..,m\right).$$

Through computing the derivative of the residuals with respect to the control coefficients *C_ij_*, the optimization problem in Equation (6) was solved via Newton's iteration method, generating highly accurate minimal surfaces compared to the conventional nodal approximation method. Using the Monge patch model, the principal curvatures and asymptotic directions can be efficiently calculated. The normal curvature of the parameterized surface states [45]:

(7) $$\kappa \;\left(\lambda \right)=\frac{L+2M\lambda +N\lambda^{2}}{E+2F\lambda +G\lambda^{2}}\;,$$

where λ  =  *dv*/*du* denotes the direction in the 2D parametric domain, *E* = ***r**_u_* · ***r**_u_*, *F* = ***r**_u_* · ***r**_v_*, *G* = ***r**_v_* · ***r**_v_* are the first fundamental forms, and *L* = ***r**_uu_* · ***n***, *M* = ***r**_uv_* · ***n***, *N* = ***r**_vv_* · ***n*** are the second fundamental forms. The asymptotic direction is defined as the direction along which the normal curvature is zero, namely *κ* (*λ*) =  0, computed as:

(8) $$\lambda_{\mathrm{LOA}}=\frac{-M\pm \sqrt{M^{2}-LN}}{N}\;.$$

Based on the definition of the principal curvature direction that the normal curvature reaches the extreme, the LOC direction is computed by *d* *κ*/*d* *λ*  =  0 as:

(9) $$\begin{array}{ccc} & & \lambda_{\mathrm{LOC}}\\ & & \;=\;\frac{-\left(EN-GL\right)\;\pm \sqrt{{\left(EN-GL\right)}^{2}-4\left(FN-GM\right)\left(EM\;-FL\right)}}{2\left(FN-GM\right)}\;. \end{array}$$

Based on the B‐spline parameterized Monge patch model, the LOA and LOC directions are generated using the finite difference method from the prescribed starting points in the parametric domain (*u*, *v*)∈[0, 1]^2^. The initial points are uniformly distributed in the *u* and *v* axes, and the successive points of the LOA and LOC are computed using Equations (8)∼(9) via the Runge‐Kutta method, until reaching the opposite domain boundaries. Due to the spatial distortion of the parametric mapping, the ribs that are spatially too close in the 3D physical domain are deleted. Given the same rib spacing, the number of deleted ribs is different for P‐ and N‐type shell lattices, which arises from their different degrees of spatial distortion, resulting in 11 and 7 rib groups remained, respectively. Since the LOA and LOC ribs are exhaustively explored in the entire design space, changing the number of rib groups is not expected to affect the conclusions of this study. The computed LOA and LOC of P‐ and N‐type TPMSs in the 2D parametric and 3D fundamental domains are shown in Figure S1b, and those in the unit cell and 1/8‐unit are shown in Figure 1. In finite element models, the meshes of shells and ribs are directly generated from the parametric surface models, which is an additional advantage as the proposed geometric model generates high‐quality quadrilateral meshes.

The locations and sizes (including the thickness and height) of ribs, as well as the shell thickness provide a large design space, complicating the design and optimization of ribbed shell lattices. In this study, we systematically investigated the effects of these design parameters on the strength and failure behavior of ribbed shell lattices. Specifically, low RDs ranging 1.0%∼4.0% were selected to explore the failure modes of TPMS thin shell lattices. The LOA and LOC ribs with varying heights and thicknesses were incorporated onto the unribbed lattices with varying RDs to seek enhancement in strength. The compressive strengths of LOA and LOC ribbed P‐ and N‐type TPMS shell lattices were evaluated by nonlinear FEA simulations and compared to those of the unribbed lattices with equal RDs.

### 4.2 Numerical Method

The nonlinear static FEA was performed under the uniaxial stress state to evaluate the strength of ribbed and unribbed shell lattices, using the commercial software ABAQUS 2024. Given the reflectional symmetry of the lattices regarding the three mid‐planes of the unit cell, the 1/8‐unit cell was utilized for analysis to save computational cost while maintaining numerical accuracy (Figures S8 and S9). The symmetric boundary conditions were imposed on the three mid‐planes, based on which periodic boundary conditions were simplified and applied to the other three end planes [7, 46]. For the clarity of illustration, the von Mises stress contour is shown in the unit cell of the lattices in the main text. The S4R shell element, a Reissner‐Mindlin plate/shell theory‐based four‐node quadrilateral shell element, was utilized for simulation. Specifically, for the analysis of ribbed lattices, the mid‐surface of the rib and that of the shell were defined as two separate parts, and the middle nodes of the rib mid‐surface were strictly tied to the mid‐surface of the shell to prevent any relative slippage in the simulation process. The tie constraints are mathematically enforced through constraint equations that modify the global stiffness matrix of the system and may thus introduce some sort of artificial stiffness, while this influence can be effectively mitigated by refining the FEA mesh. For the simulation of ribbed and unribbed P‐type shell lattices, a total number of 2 400∼5 500 quadrilateral elements were generated within the 1/8‐unit cell, in which the ratio of the average side length (*Δ*) of each element to the unit cell size (*D*), namely *Δ*/*D*, ranges between 4.34 × 10^−3^ and 1.25 × 10^−2^, with a mean value of 1.14 × 10^−2^. In comparison, a total number of 10 000∼20 000 quadrilateral elements in the 1/8‐unit cell were utilized for the simulation of N‐type shell lattices, in which the ratio of average side length to unit cell size (*Δ*/*D*) ranges between 7.15 × 10^−3^ and 3.58 × 10^−2^, with a mean value of 1.21 × 10^−2^. The FEA mesh is sufficiently precise to obtain accurate numerical results, as validated by a detailed mesh convergence analysis (Figure S7), and the 0.2% offset strength is defined as the compressive strength of the lattices. The uniaxial tensile test of standard tensile samples made by µLPBF was conducted to determine the stress‐strain curve of the SS316L constitutive materials, based on the ASTM E8/E8M standard, under a constant nominal strain rate of 0.001 s^−1^ [22]. The test reveals the Young's modulus (*E_s_* = 189310.0 MPa), Poisson's ratio (*ν_s_* = 0.3), and uniaxial yield strength (*S_y_* = 520.0 MPa) of the SS316L materials, which, together with their true stress versus plastic strain curve (Figure S14), were utilized in the nonlinear simulation of the ribbed and unribbed shell lattices.

### 4.3 Fabrication Method

The P‐type TPMS shell lattices, including unribbed, LOA ribbed, and LOC ribbed lattices with an RD of 2.03%, were manufactured to validate the rib reinforcement effect via quasi‐static compression tests. Traditional manufacturing techniques, such as casting, forging, welding, injection molding, and CNC milling, face significant challenges due to the complexity of micro‐architectures. Accordingly, additive manufacturing is selected as the fabrication method to ensure both the fabrication quality and efficiency. The samples were fabricated by an in‐house Hans' Laser M100 machine, a high‐precision µLPBF platform equipped with a Ytterbium laser source whose beam diameter and wavelength are 25 and 1.07 µm, respectively. The samples were made in austenitic SS316L materials whose powder size ranges from 5 to 25 µm, with a median particle size (D_50_) of 16.27 µm. The unribbed, LOA ribbed, and LOC ribbed lattices with a 3 × 3 × 3 array of unit cells were fabricated along the [001] direction, and three samples were made for each lattice to validate the experimental repeatability. To ensure the fabrication quality, the rib thickness was initially set to match the shell thickness. The hanging ribs beneath the shell surface were then removed to prevent printing failure, and the thickness of the remaining ribs was appropriately increased to ensure an identical total mass of the samples. A novel single‐track scanning strategy was adopted in the fabrication process [22, 40], in which the shell thickness was fixed at 70 µm, and the unit cell size was adjusted to achieve the target RD. This strategy enables to fabricate ultralight metallic lattices with an RD around or lower than 2.0% [22, 40], lower than those in most existing studies, within which the rib reinforcement effect of ultralight shell lattices via buckling suppression can be validated experimentally. The hatch distance, scanning speed, layer thickness, and laser power were taken as 50 µm, 1000 mm/s, 10 µm, and 50 W, respectively, and the patterns between successive layers were rotated by a hatch angle of 67° to reduce the thermal stress. These parameters were optimized in our prior study, revealing dense and pore‐free internal micro‐architectures for the as‐printed samples [35]. Following the completion of fabrication, the samples were removed from the building plate using electrical discharge machining, and the ultrasonic vibration was used to clean the samples in ethanol. Moreover, as the SS316L samples exhibit superior strength and ductility for the solidification‐enabled micro‐architectures of dislocations [35, 47, 48], and the heat treatment procedure tends to decrease their mechanical properties [49], the as‐fabricated samples were not heat‐treated in this study.

### 4.4 Characterization Method

The RD of the sample was evaluated based on its actually measured mass, the bounding box size, and the density of SS316L (*ρ*_*s*_ =  7.98 g/cm^3^). The Scanco Medical µCT‐35, a micro‐CT equipped with a 70 kV X‐ray source, was used to characterize the manufacturing fidelity of the samples. Afterwards, quasi‐static compression tests were conducted on an MTS Model 370.10 machine to evaluate the Young's moduli, strengths, NPS, SEA, stress‐strain curves, and compressive deformation behaviors of the samples. The Young's moduli were evaluated as the average slope of the linear stage of 3 unload curves from the load‐unload repetitive compression tests, and the effect of machine stiffness was eliminated to avoid underestimating the Young's moduli of the samples [7]. The 0.2% offset strength was evaluated as the compressive strength of samples in large‐strain compression tests under a nominal strain rate of 0.001 s^−1^ [6, 22]. The NPS and SEA were calculated based on the experimentally measured stress‐strain curves of the samples [7]. Three repeated test samples were used to validate the experimental repeatability, and the error bars in the experimental data were utilized to illustrate the variation in mechanical performances. In addition, the von Mises strain distributions of the samples were further evaluated based on the experimentally captured deformation patterns, using the 2D DIC software VIC‐2D (Correlated Solutions, US).

## Author Contributions

Winston Wai Shing Ma: Writing – original draft, Writing – review & editing, Methodology, Investigation, Validation. Lei Zhang: Writing – review & editing, Methodology, Investigation. Junhao Ding: Methodology, Investigation, Validation. Shuo Qu: Methodology, Investigation, Validation. Michael Yu Wang: Writing – review & editing, Supervision, Conceptualization. Xu Song: Writing – review & editing, Supervision, Funding acquisition, Conceptualization. Ming Wang Fu: Writing – review & editing, Supervision, Funding acquisition, Conceptualization.

## Conflicts of Interest

The authors declare no conflicts of interest.

## Supporting information




**Supporting File**: advs73802‐sup‐0001‐SuppMat.docx.

**Supporting File**: advs73802‐sup‐0002‐SuppMat.xlsx.

## Data Availability

The data that support the findings of this study are available from the corresponding author upon reasonable request.
