# Supplementary material for: Rib‐Reinforced Ultralight and Ultra‐Strong Shell Lattices
Source: Adv Sci (Weinh). 2026 Jan 21;13(17):e18357. doi: 10.1002/advs.202518357 (PMC13042649; doi:10.1002/advs.202518357)
Supplement: Supplementary file 1 — Supporting File: advs73802‐sup‐0001‐SuppMat.docx. [file ADVS-13-e18357-s002.docx]

Supporting Information

**Rib-reinforced ultralight and ultra-strong shell lattices**

Winston Wai Shing Ma ^†^, Lei Zhang ^†^, Junhao Ding, Shuo Qu, Michael Yu Wang, Xu Song ^*^, Ming Wang Fu ^*^

^†^ These authors contributed equally: Winston Wai Shing Ma, Lei Zhang.

^*^ Corresponding authors: Xu Song (xsong@cuhk.edu.hk), Ming Wang Fu (mmmwfu@polyu.edu.hk).

**List of abbreviations:**

- Relative densities (RDs)
- Triply periodic minimal surface (TPMS)
- Line of asymptotes (LOA)
- Line of principal curvatures (LOC)
- Primitive (P)
- Finite element analysis (FEA)
- Neovius (N)
- Micro laser powder bed fusion (µLPBF)
- Stainless steel 316 L (SS316L)
- Digital image correlation (DIC)
- Normalized plateau stresses (NPSs)
- Specific energy absorption (SEA)
- Building direction (BD)
- Stereolithography (STL)
- Two-dimensional (2D)
- Partial differential equation (PDE)
- Three-dimensional (3D)
- Periodic boundary conditions (PBCs)
- Degrees of freedom (DOFs)

# B-spline parameterized Monge patch models of Primitive (P)- and Neovius (N)-type triply periodic minimal surfaces (TPMSs)

This section illustrates the geometric modelling processes and results of P- and N-type TPMSs. As shown in **Fig. S1**a, the fundamental quadrirectangular tetrahedra of both surfaces are identical. The vertices of the quadrirectangular tetrahedron are $\text{O}(0,0,0)$, $\text{A}(\frac{1}{2},0,0)$, $\text{B}(\frac{1}{2},\frac{1}{2},0)$, and $\text{C}(\frac{1}{2},\frac{1}{2},\frac{1}{2})$. However, the mappings from the two-dimensional (2D) parametric domain to the three-dimensional (3D) physical domain are different. For P-family surfaces, *u* and *v* axes correspond to OA and BC axes, i.e., $\overline{\text{OU}}=u\cdot\overline{\text{OA}}=(\frac{u}{2},0,0)$, $\overline{\text{BV}}=v\cdot\overline{\text{BC}}=(0,0,\frac{v}{2})$; while for N-family surfaces, *u* and *v* axes correspond to OB and AC axes, i.e., $\overline{\text{OU}}=u\cdot\overline{\text{OB}}=(\frac{u}{2},\frac{u}{2},0)$, $\overline{\text{AV}}=v\cdot\overline{\text{AC}}=(0,\frac{v}{2},\frac{v}{2})$. The difference in mapping results in different topologies of P- and N-type TPMSs, as shown in Fig. S1a. By solving the optimization problem in Eq. (6), the minimal surface in the fundamental domain (1/48-unit cell) was obtained (shaded in red). Through successive mirror operations, the 1/8-unit cell and unit cell of TPMSs were generated (Fig. S1a). The B-spline parameterized Monge patch models map a 2D parametric domain to a 3D surface, generating high-quality quadrilateral meshes that can be used directly in finite element analysis (FEA).

Based on the Monge patch models, the principal curvatures and asymptotic directions of P- and N-type TPMSs at any point were calculated using Eqs. (7)~(9). To generate a line of asymptotes (LOA) or a line of principal curvatures (LOC), starting points uniformly distributed on the boundary of the parametric domain were selected, and the finite difference method was used to compute the successive points on the LOA or LOC until reaching the opposite domain boundary. In this work, the interval of the starting points was set as 0.1 along the *u* and/or *v* axes. For the P-type TPMS, 11 groups of ribs were generated. In contrast, for the N-type TPMS, the LOA and LOC with the starting points of *u*≤0.5 (on the bottom boundary line, *v*=0) and *v*≥0.5 (on the right boundary line, *u*=1) were deleted because these lines are spatially too close, thereby resulting in 7 groups of ribs remaining. The LOA and LOC of P- and N-type TPMSs within the 2D parametric and 3D fundamental domains are shown in Fig. S1b, which can be utilized to generate the LOA/LOC within the 1/8-unit cell and unit cell via mirror operations. Afterwards, the ribs were generated by extruding these lines along their normal directions, as shown in Fig. 1f~i.


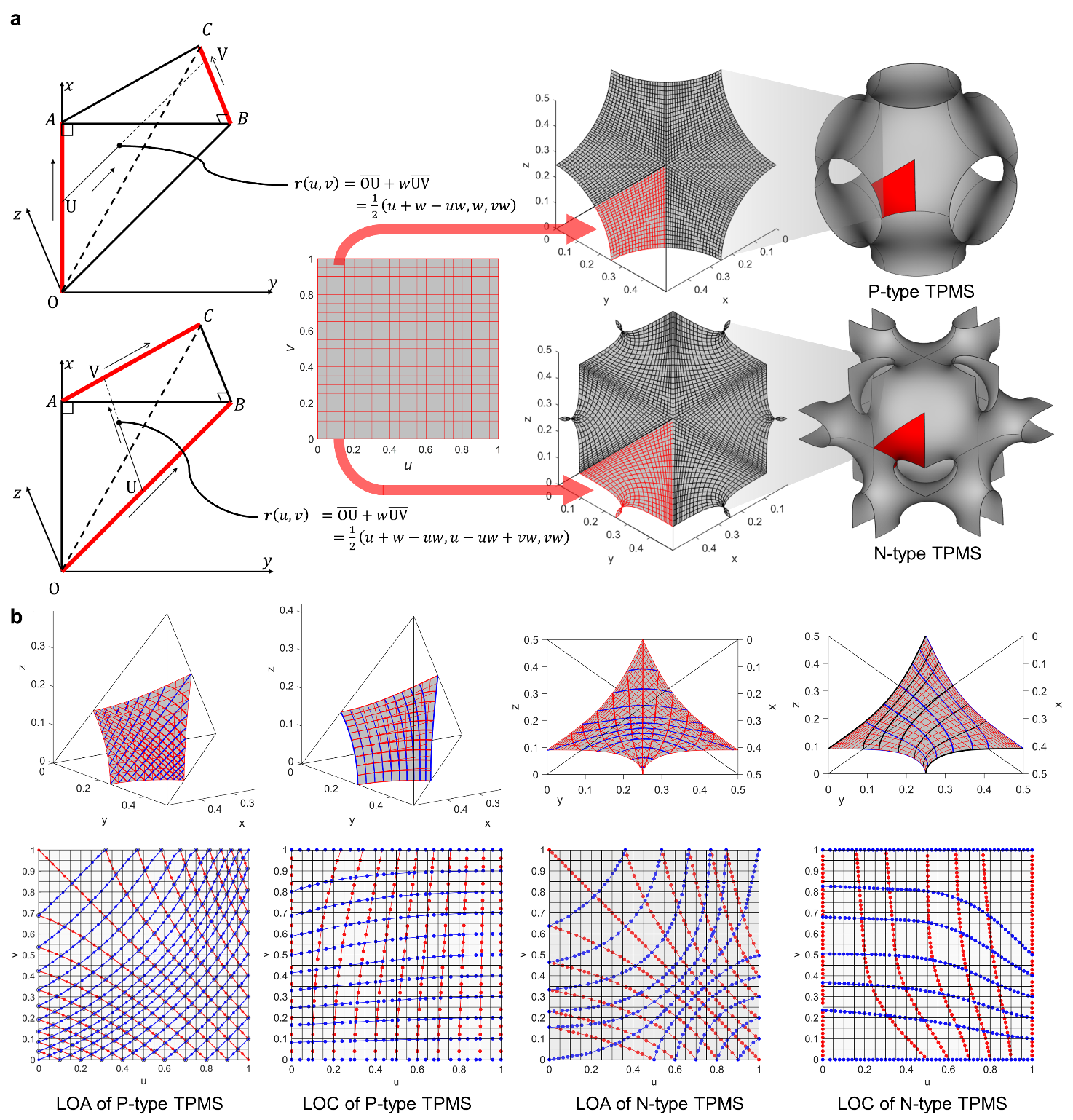


**Fig. S1. The B-spline parameterized Monge patch models of P- and N-type TPMSs.** (a) The P- and N-type TPMSs constructed via the proposed parametric models, and (b) their LOAs and LOCs in the 3D fundamental and 2D parametric domains.

# Design guideline of ribbed shell lattices based on the membrane theory of thin shells

The membrane theory of thin shells was utilized as a design guideline for the rib-reinforced shell lattices. According to the fundamental equations of the membrane theory of thin shells [1], the two normal stress resultants *N*_1_ and *N*_2_ satisfy $N_{1}\kappa_{1}+N_{2}\kappa_{2}=0$, in which *κ*_1_ and *κ*_2_ denote the two normal curvatures of the shell mid-surface in any orthogonal local coordinates. In the LOC coordinates of TPMSs, the two normal curvatures are equal to the principal curvatures and satisfy the relation $\kappa_{1}^{(\text{LOC})}+\kappa_{2}^{(\text{LOC})}=0$, implying $N_{1}^{(\text{LOC})}=N_{2}^{(\text{LOC})}$ as well as a maximum shear stress state. According to Mohr's circle, the shear stress vanishes in the LOA coordinates, since the LOA coordinates have an angle of $45^{\circ}$ to the LOC coordinates (**Fig. S2**a), implying a principal stress state in LOA coordinates. Therefore, the incorporation of LOA ribs tends to achieve greater strength enhancement than LOC ribs.

To validate the applicability of using the membrane theory of thin shells as the design guideline, a linear elastic FEA was performed on the 1/8-unit cell of P-type TPMS shell lattices with a relative density (RD) of 2.5% under the uniaxial strain loading. The ABAQUS STRI3 shell element was adopted for analysis, based on the classical Kirchhoff-Love plate/shell theory. The numerically evaluated stress state in the ABAQUS default local coordinate system was converted to the LOA and LOC coordinates via coordinate transformation. The stress distributions show that the shear stress *S*_12_ vanishes in the majority of regions in the LOA coordinates, except for the region near the umbilics, where the curvature directions are indeterminate (Fig. S2b). Similarly, the two normal stress components *S*_11_ and *S*_22_ are approximately equal to each other in most regions of the lattices in the LOC coordinates, except for the umbilical region. These findings indicate that most regions of the lattices are in a state of principal shear stress (Fig. S2c), consistent with the predictions of the membrane thin-shell theory. Furthermore, the angular deviations between the LOA and principal stress directions at the Gaussian point of each quadrilateral element under the uniaxial strain and pure shear stress states are further evaluated. The rendered angular deviation plots indicate that most elements exhibit low angular deviations under both load cases (Fig. S2d). The statistical analysis demonstrates that the angular deviations of the 73.5% and 85.7% elements are less than 15° under uniaxial strain and pure shear loads, respectively, indicating that the LOA and principal stress directions are highly aligned across most regions of the TPMS shin shell lattices. Overall, the membrane theory of thin shells is shown to hold in most regions, and can thus be adopted as a design guideline for rib-reinforced TPMS shell lattices.


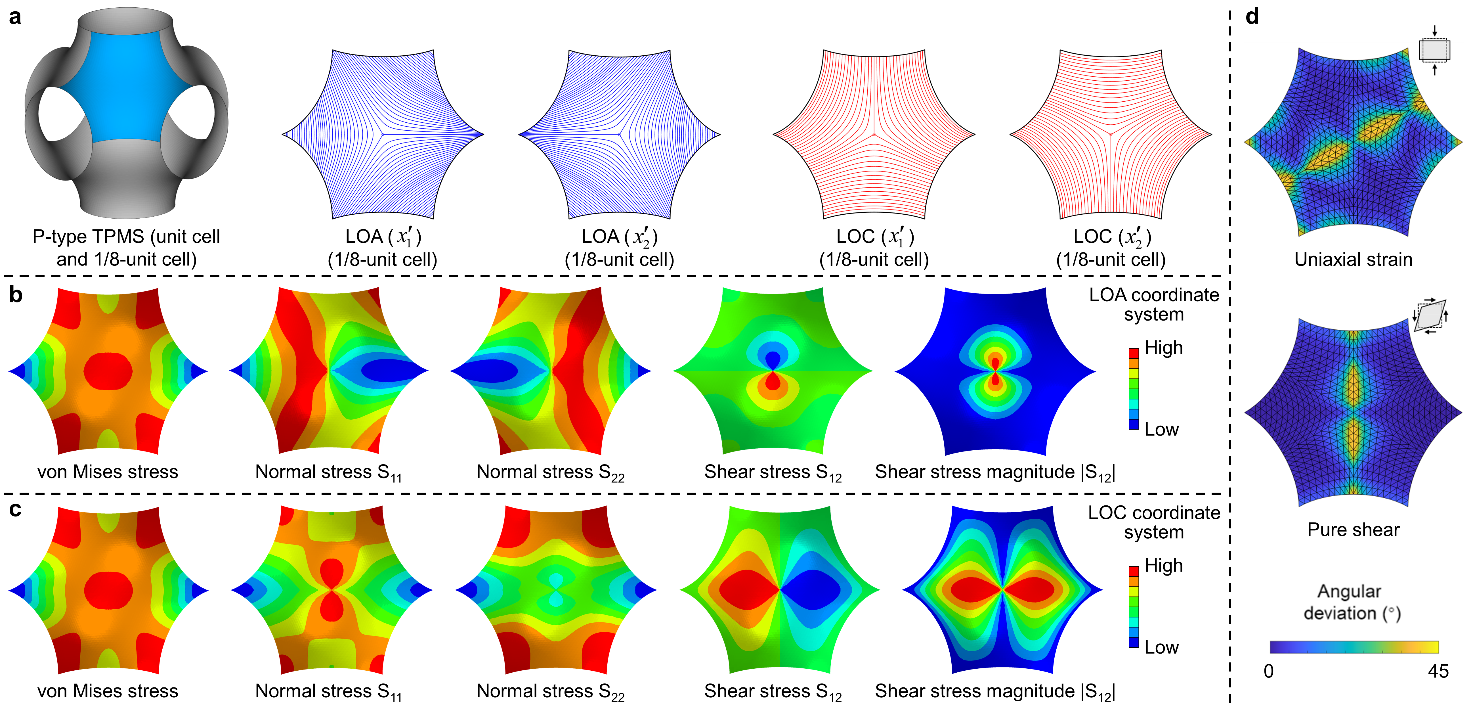


**Fig. S2****. Validation of the applicability of the membrane theory of thin shells as the design guideline for P-type TPMS shell lattices.** (a) The LOA and LOC of P-type TPMS; the distributions of von Mises stress, normal and shear stress components, and shear stress magnitude of P-type TPMS thin shell lattices under the uniaxial stress state in the (b) LOA and (c) LOC coordinates; and (d) the angular deviation of the LOA and principal stress directions of P-type TPMS shell lattices under the uniaxial strain and pure shear stress states, respectively.

# Design of LOA and LOC ribbed P- and N-type TPMS shell lattices

Representative layouts of LOA and LOC ribbed P- and N-type TPMS shell lattices are explored to study the effects of ribs on the strength and failure behaviors of ribbed shell lattices (**Fig. S3**a-d). At any point on the TPMS, there is an angle of $45^{\circ}$ between the principal curvature and asymptotic directions, as illustrated in Fig. S2a. In total, we have orchestrated 11 layouts of LOA ribbed P-type shell lattices (Fig. S3a), 11 layouts of LOC ribbed P-type shell lattices (Fig. S3b), 7 layouts of LOA ribbed N-type shell lattices (Fig. S3c), and 7 layouts of LOC ribbed N-type shell lattices (Fig. S3d) for analysis, respectively. The unribbed lattices with four different RDs of 1.0%, 1.5%, 2.0%, and 2.5% were selected for the design of ribbed lattices, and the rib thickness was taken as 0.5, 1.0, and 2.0 times the shell thickness, respectively. Additionally, the rib height was treated as a design variable, and ribbed lattices with heights ranging from 0.05 to 0.2 times the unit cell size were considered for several representative ribs. The compressive strength of the ribbed lattices was evaluated by nonlinear FEA simulations and compared to that of their unribbed counterparts with equal RDs.


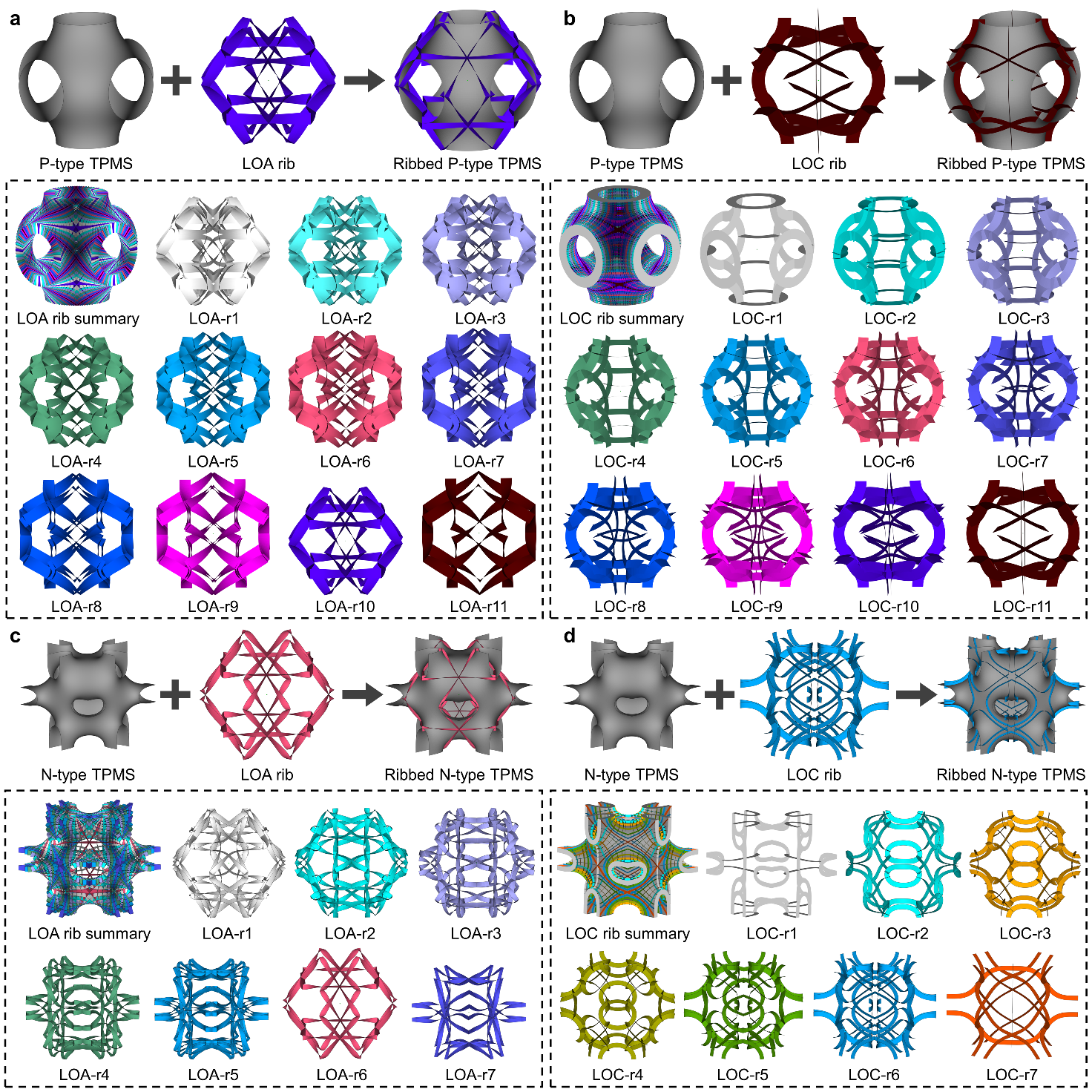


**Fig. S3. Representative designs of incorporating LOA and LOC ribs onto P- and N-type TPMS shell lattices.** (a) The ribbed P-type shell lattices with 11 layouts of LOA ribs, and (b) those with 11 layouts of LOC ribs. (c) The ribbed N-type shell lattices with 7 layouts of LOA ribs, and (d) those with 7 layouts of LOC ribs.

# Numerical results of P-type TPMS shell lattices

## Linear buckling and nonlinear static analyses of P-type TPMS shell lattices

The numerical results of the linear buckling and nonlinear static analyses of P-type TPMS shell lattices with RDs ranging 1.0%~4.0% are shown in **Fig. S4**. The normalized strength (including normalized critical buckling strength and normalized 0.2% offset strength) versus RD plot reveals that P-type shell lattices undergo a failure mode transition from material yielding to micro-architecture buckling in the 1.5%~2.0% RD range (Fig. S4a-b). The transition is manifested as a sudden change in slope of the normalized 0.2% offset strength versus RD plot in logarithmic coordinates [2] (Fig. S4a). Besides, the transition is further validated by a significant difference in the deformation state and von Mises stress distribution of the lattices with different RDs (Fig. S4b). More specifically, the boundary edge of the lattice with 1.5% RD undergoes evident distortional deformations, indicating a shell buckling failure mode. In contrast, no significant distortions occur in the boundary edge of the lattice with 2.0% RD, implying a failure mode dominated by bending deformations and material yielding. To further illustrate this point, the critical buckling modes and displacement magnitude distributions of P-type shell lattices with different RDs are shown in Fig. S4c. The critical buckling mode reveals that the central region near the umbilical point bulges out and the edge region near the boundary undergoes severe distortional deformations (Fig. S4c), matching well with the deformed state of the lattices with RDs ranging 1.0%~1.5% in the nonlinear simulation (Fig. S4b). This comparison further demonstrates the yielding-to-buckling failure mode transition of P-type TPMS shell lattices in the 1.5%~2.0% RD range.


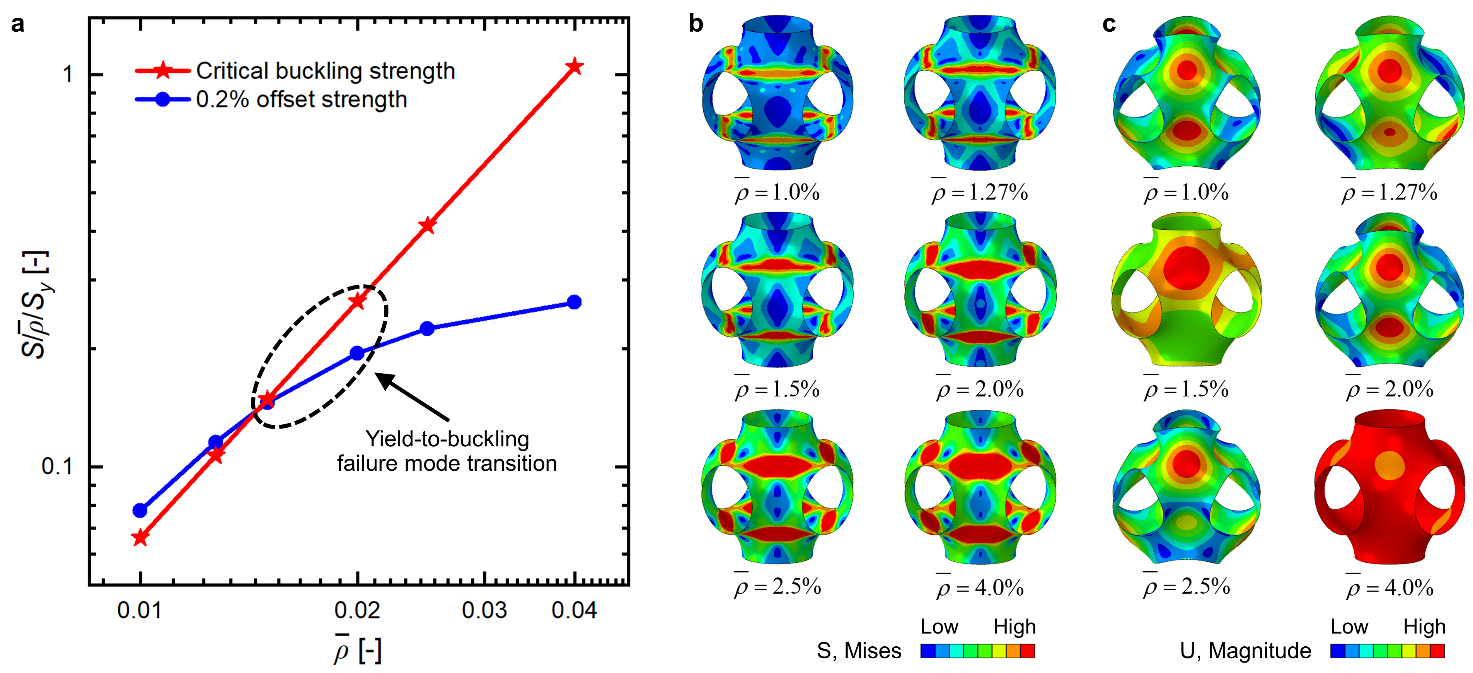


**Fig. S4. Linear buckling and nonlinear static analyses of P-type TPMS shell lattices with RDs ranging 1.0%~4.0%.** (a) Normalized strength versus RD plots of P-type shell lattices with varying RDs, including the normalized critical buckling strength and the normalized 0.2% offset strength. (b) The von Mises stress distributions at the 0.2% offset point and (c) the critical buckling modes of P-type shell lattices with varying RDs.

## Numerical results of ribbed P-type TPMS shell lattices

The numerically evaluated strength, stress-strain curves, and von Mises stress distributions of four representative layouts of LOA ribbed P-type shell lattices with varying RDs and their unribbed counterparts with equal RDs are shown in **Fig. S5**a-h. Among the four layouts of ribbed lattices, LOA-r1 (Fig. S5a) and LOA-r10 (Fig. S5c) ribbed lattices can achieve enhanced strength than their unribbed counterparts, while LOA-r5 (Fig. S5b) and LOA-r11 (Fig. S5d) ribbed lattices exhibit lower strength. The strength of LOA-r1 and LOA-r10 ribbed lattices shows an overall increasing trend with the decrease of RDs, in which LOA-r10 ribbed lattices possess higher strength than LOA-r1 ribbed lattices. In particular, the LOA-r10 ribbed lattice can achieve 62.0% strength enhancement at 1.36% RD, while the LOA-r1 ribbed lattice achieves 14.7% enhancement at 1.68% RD. The enhancement or decrease in strength are further illustrated by their stress-strain curves and von Mises stress distributions at the 0.2% offset point (Fig. S5e-h). More specifically, the LOA-r10 ribs directly pass through the umbilical point and is able to suppress the shell buckling deformation, thus resulting in a significant enhancement in strength. The strengthening effect is further validated by the high stress state of ribs in Fig. S5g, in which the ribs efficiently bear loads and is in a high stress state. In contrast, the LOA-r1 ribs do not directly pass through the umbilical point, which leads to lower strength enhancement (Fig. S5e). Even worse, incorporating LOA-r5 and LOA-r11 ribs turns out to decrease in strength, as a result of their inability to suppress the shell buckling deformation, which is demonstrated by the low stress state of the ribs (Fig. S5f and h). Overall, incorporating appropriate rib layouts can effectively enhance the strength of ultralight P-type shell lattices by tuning their micro-architecture buckling deformation.

By changing the rib height, a series of LOA-r10 ribs were generated further to improve the strength of the ribbed shell lattices (Fig. S5i). The strength of the ribbed lattices and their unribbed counterparts is shown in Fig. S5j, k, and l, and their stress-strain curves and von Mises stress distributions are illustrated in Fig. S5m, n, and o, respectively. The ribbed lattices exhibit similar stress distribution patterns, in which the magnitude of strength enhancement shows a slight decreasing trend with an increase in height. More specifically, the LOA-r10-h1 ribbed lattice (Fig. S5j) achieved 70.2% strength enhancement at 1.18% RD, the LOA-r10-h2 ribbed lattice (Fig. S5k) got 67.9% enhancement at 1.27% RD, and the LOA-r10-h4 ribbed lattice (Fig. S5l) had 52.5% enhancement at 1.46% RD, with the rib thickness equal to 0.5 times the shell thickness. The magnitude of strength enhancement was increased with decreasing RDs, implying a more significant buckling tuning effect at lower RDs. The raw data of the normalized strength of the 11 layouts of LOA ribbed P-type shell lattices are listed in the Supplementary File "Original Data.xlsx".


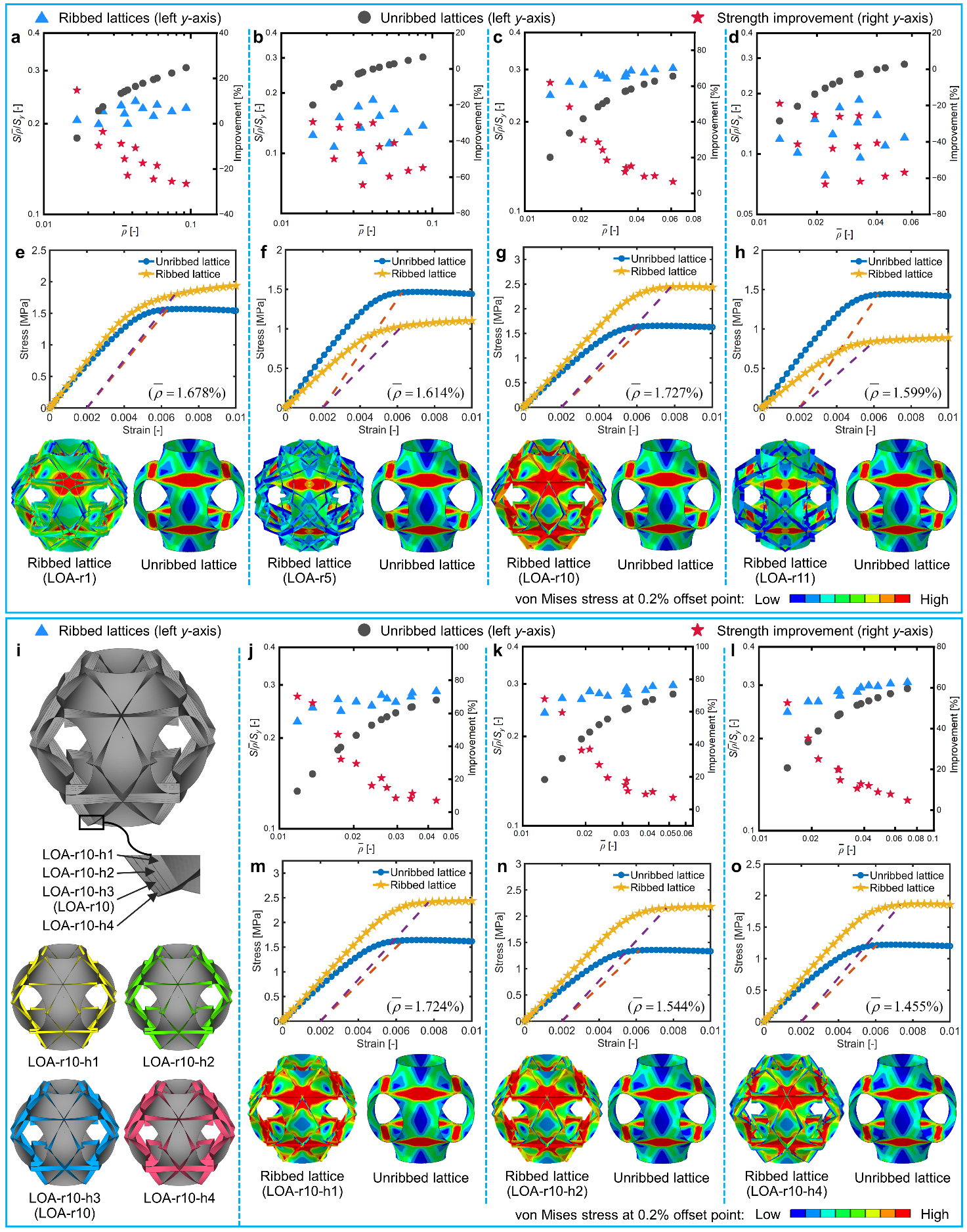


**Fig. S5. Simulation results of four representative layouts of LOA ribbed P-type TPMS shell lattices and their unribbed counterparts with equal RDs.** The strength (S) normalized by the yield strength of the constitutive material (S_y_) versus RD ($\overline{\rho}$) plots of (a) LOA-r1, (b) LOA-r5, (c) LOA-r10, and (d) LOA-r11 ribbed lattices and their unribbed counterparts, and (e-h) their stress-strain curves and von Mises stress distributions at the 0.2% offset point, as compared to their unribbed counterparts. (i) The LOA-r10 ribs with varying heights, the normalized strength versus RD plots of (j) LOA-r10-h1, (k) LOA-r10-h2, and (l) LOA-r10-h4 ribbed lattices and their unribbed counterparts, and (m-o) their stress-strain curves and von Mises stress distributions at the 0.2% offset point, as compared to their unribbed counterparts.

Besides, the incorporation of LOC ribs to seek the strength enhancement of P-type TPMS shell lattices is further studied. Among the 4 layouts of LOC ribbed lattices, the LOC-r1 (**Fig. S6**a) and LOC-r5 (Fig. S6b) ribbed lattices possess lower strength than their unribbed lattices of equal RDs, while incorporating LOC-r10 (Fig. S6c) and LOC-r11 (Fig. S6d) ribs can achieve enhanced strength. The strength enhancement or decrease is further illustrated by their stress-strain curves and von Mises stress distributions in Fig. S6e-h. More specifically, the incorporation of the LOC-r11 rib that directly passes through the umbilical point can enable a higher strength enhancement than that of the LOC-r10 rib. In particular, the LOC-r11 ribbed lattice can achieve 112.3% strength enhancement at 1.28% RD, while the LOC-r10 ribbed lattice achieves 26.4% enhancement at 1.53% RD.

The LOC-r11 ribs with varying heights were explored to improve the strength of the ribbed lattices further (Fig. S6i). The strength of the ribbed lattices and their unribbed counterparts is shown in Fig. S6j, k, and l, and their stress-strain curves and von Mises stress distributions are illustrated in Fig. S6m, n, and o, respectively. The ribbed lattices exhibit similar stress distribution patterns, in which the ribs effectively bear loads by suppressing the shell buckling deformation and are in a high stress state. Among the four ribbed lattices, the LOC-r11-h2 ribbed lattice can achieve the highest enhancement in strength. In particular, the LOC-r11-h1 ribbed lattice (Fig. S6j) achieves 77.8% strength enhancement at 1.14% RD, the LOC-r11-h2 ribbed lattice (Fig. S6d) achieves 112.3% strength enhancement at 1.28% RD, the LOC-r11-h3 ribbed lattice (Fig. S6k) achieves 51.0% enhancement at 1.42% RD. The LOC-r11-h4 ribbed lattice (Fig. S6l) achieves 38.2% enhancement at 1.57% RD, with the rib thickness equal to 0.5 times the shell thickness. The raw data of the normalized strength of the 11 layouts of LOC ribbed P-type shell lattices are listed in the Supplementary File "Original Data.xlsx".


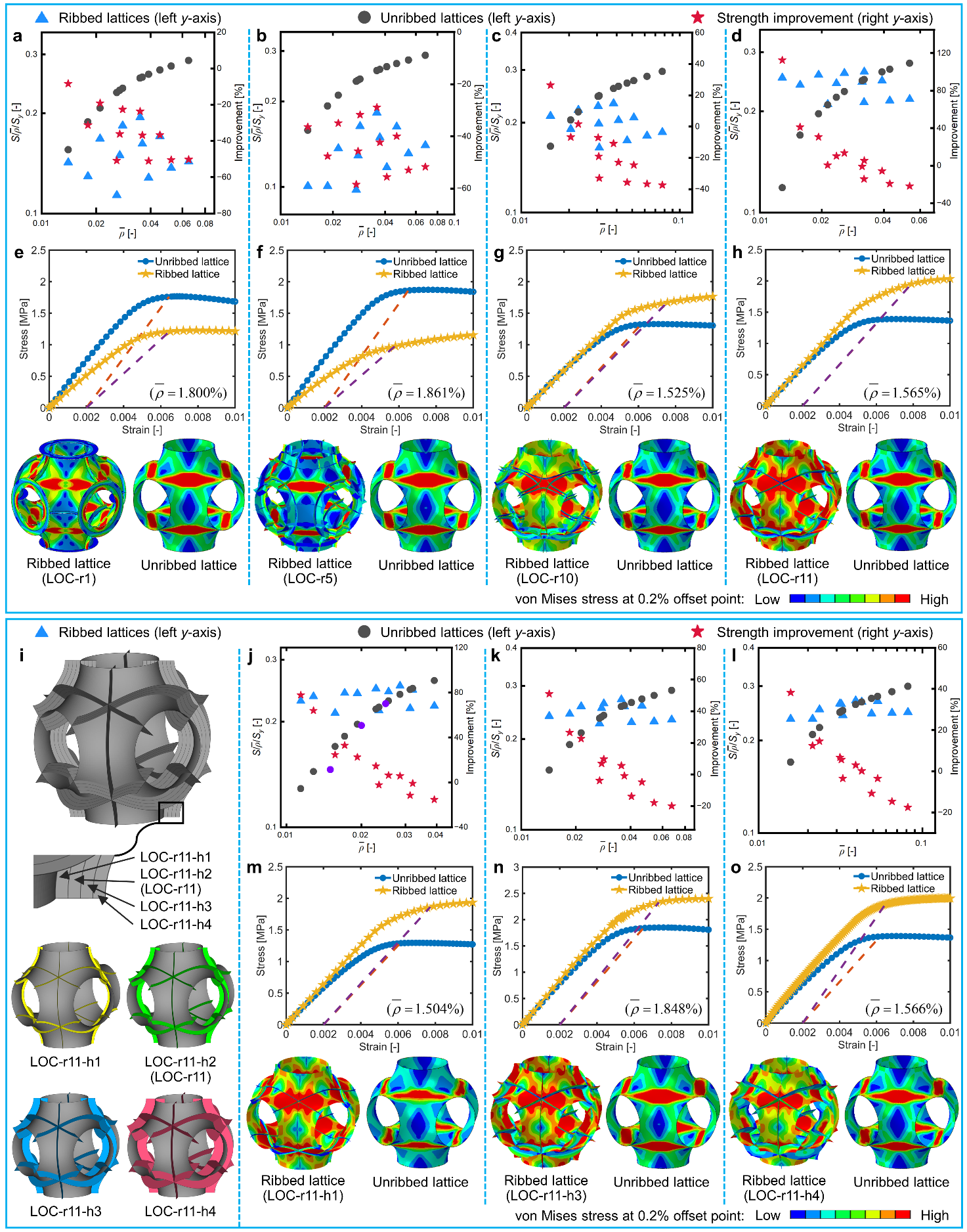


**Fig. S6. Simulation results of four representative layouts of LOC ribbed P-type TPMS shell lattices and their unribbed counterparts with equal RDs.** The normalized strength versus RD plots of (a) LOC-r1, (b) LOC-r5, (c) LOC-r10, and (d) LOC-r11 ribbed lattices and their unribbed counterparts, and (e-h) their stress-strain curves and von Mises stress distributions at the 0.2% offset point, as compared to their unribbed counterparts. (i) The LOC-r11 ribs with varying heights, the normalized strength versus RD plots of (j) LOC-r11-h1, (k) LOC-r11-h3, and (l) LOC-r11-h4 ribbed lattices and their unribbed counterparts, and (m-o) their stress-strain curves and von Mises stress distributions at the 0.2% offset point, as compared to their unribbed counterparts.

# Mesh convergence analysis of numerical results

A mesh convergence analysis is conducted to further validate the numerical results of this study (**Fig. S7**). By increasing or decreasing the average side length (*Δ*) of quadrilateral elements, two additional meshes are generated, namely the "coarse mesh" and "fine mesh", while the original mesh is referred to as the "medium mesh". The numbers (*N*) of quadrilateral elements in the coarse and fine meshes are controlled to be 0.50~0.67 and 1.5~2.0 times that of the medium mesh, respectively. Based on the three sets of meshes, the mechanical properties, including the von Mises stress distribution (under a macro compressive strain of 0.01), Young's modulus, and strength, of three representative lattices, including the unribbed (Fig. S7a and d), LOA-r10-h1 ribbed (Fig. S7b and e), and LOC-r11-h1 ribbed (Fig. S7c and f) P-type TPMS shell lattices, are numerically evaluated. These lattices share an equal RD of 1.75%, and the rib thickness is set to be the same as the shell thickness for the LOA-r10-h1 and LOC-r11-h1 ribbed lattices. The numerical results reveal that the relative differences in Young's modulus and strength evaluated using the coarse and medium meshes are less than 2.0%, whereas those evaluated using the medium and fine meshes are less than 1.0%. Therefore, the numerical results evaluated using the original medium mesh are justified to be accurate enough, and the mesh convergence criteria are set as "increasing the number of quadrilateral elements by 1.5~2.0 times results in a relative change of lower than 1% for the numerically evaluated Young's modulus and strength" accordingly.


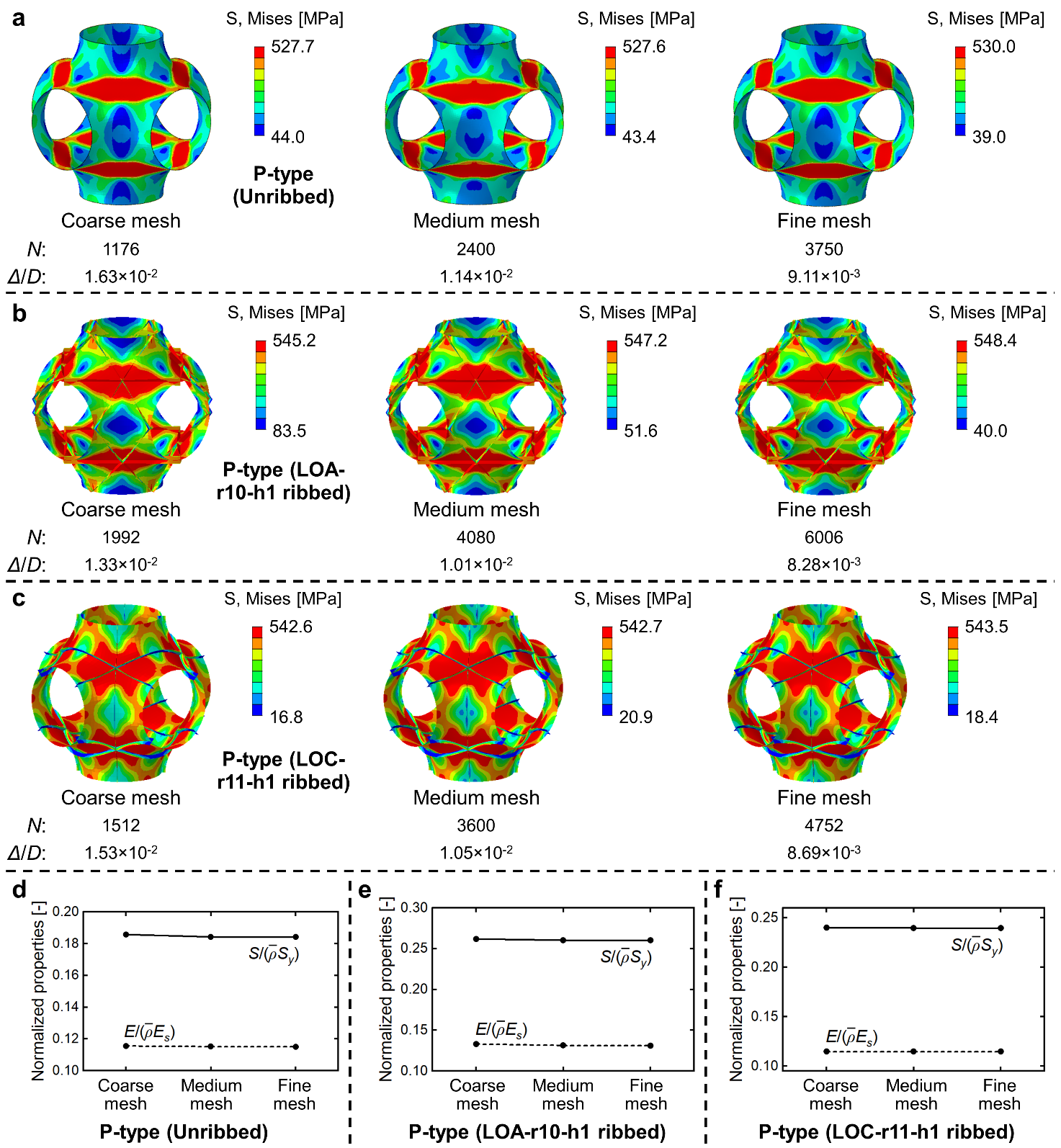


**Fig. S7. Mesh convergence analysis results of the unribbed, LOA-r10-h1 ribbed, and LOC-r11-h1 ribbed P-type TPMS shell lattices.** The numerically evaluated von Mises stress distribution (under a macro compressive strain of 0.01) of (a) the unribbed, (b) LOA-r10-h1 ribbed, and (c) LOC-r11-h1 ribbed P-type shell lattices using the coarse, medium, and fine meshes, and the comparison between the numerically evaluated normalized Young's modulus ($E/\overline{\rho}/E_{s}$) and strength ($S/\overline{\rho}/S_{y}$) of (d) the unribbed, (e) LOA-r10-h1 ribbed, and (f) LOC-r11-h1 ribbed lattices. In the plots, N denotes the number of quadrilateral elements within the 1/8-unit cell, and Δ/D denotes the ratio of the average side length (Δ) of quadrilateral elements to the unit cell size (D).

# Validation of numerical results obtained using the 1/8-unit cell with simplified boundary conditions

For the numerical simulation of periodic lattices, the unit cell is typically adopted for analysis, in which periodic boundary conditions (PBCs) are imposed on the corresponding node pairs of opposite end planes to reflect the periodicity (**Fig. S8**) [3]:

$$\begin{matrix} u_{i}^{+}-u_{i}^{-}=\varepsilon_{ij}(X_{j}^{+}-X_{j}^{-}),\text{ }(i=1,2,3), \\ \theta_{i}^{+}-\theta_{i}^{-}=0,\text{ }(i=1,2,3), \end{matrix}$$

in which the superscripts + and – represent the corresponding nodes on opposite end planes, *j* is a dummy index using the Einstein notation, ***u*** and ***θ*** denote the translational and rotational degrees of freedom (DOFs), respectively, and ***X*** denotes the node locations. In some common cases, the lattices are reflectionally symmetric regarding the three middle planes of the unit cell, and the PBCs can be further simplified by considering the reflectional symmetry of lattices. For the uniaxial stress state considered in this study, the symmetric boundary conditions should be imposed on the three middle planes. More specifically, the three DOFs *u*_1_, *θ*_2_, and *θ*_3_ should vanish on the middle plane *x*_0_ (Fig. S8), otherwise the deformation of lattices cannot maintain symmetry herein [3, 4]. Similarly, the three DOFs *u*_2_, *θ*_1_, and *θ*_3_ should vanish on the middle plane *y*_0_, and the three DOFs *u*_3_, *θ*_1_, and *θ*_2_ should vanish on the middle plane *z*_0_ (Fig. S8). Based on this, the 1/8-unit cell can be adopted for the FEA, in which the PBCs in Eq. can be simplified as [3, 4]:

$$\begin{matrix} u_{1}^{x_{0}}=0,\text{ }\theta_{2}^{x_{0}}=0,\text{ }\theta_{3}^{x_{0}}=0, \\ u_{1}^{x_{+}}=\varepsilon_{11}\frac{D}{2},\text{ }\theta_{2}^{x_{+}}=0,\text{ }\theta_{3}^{x_{+}}=0, \\ u_{2}^{y_{0}}=0,\text{ }\theta_{1}^{y_{0}}=0,\text{ }\theta_{3}^{y_{0}}=0, \\ \theta_{1}^{y_{+}}=0,\text{ }\theta_{3}^{y_{+}}=0, \\ u_{3}^{z_{0}}=0,\text{ }\theta_{1}^{z_{0}}=0,\text{ }\theta_{2}^{z_{0}}=0, \\ \theta_{1}^{z_{+}}=0,\text{ }\theta_{2}^{z_{+}}=0, \end{matrix}$$

in which *ε*_11_ denotes the normal strain along *x* direction, and *D* denotes the unit cell size. In addition to the simplified boundary conditions in Eq. , two extra constraints need to be further imposed via the kinematic coupling or linear equation constraint in ABAQUS to ensure the periodicity of deformation. Specifically, one constraint should be imposed to enable identical DOF *u*_2_ for all nodes on the end plane *y*_+_, and the other constraint should be imposed to enable identical DOF *u*_3_ for all nodes on the end plane *z*_+_.


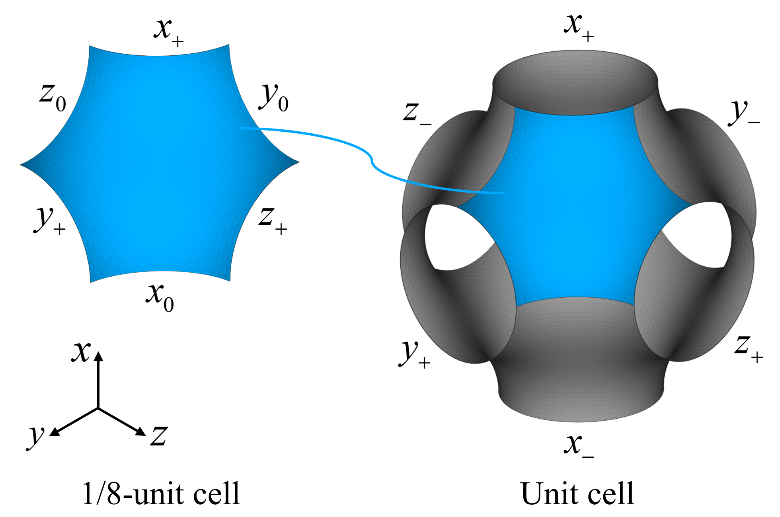


**Fig. S8. Schematic of simplified boundary conditions on the 1/8-unit cell of P-type TPMS shell lattices.**

Moreover, a comparison of the mechanical properties, including the Young's modulus, strength, and von Mises stress distribution, of P- and N-type TPMS shell lattices with 1.75% RD is made to demonstrate the correctness of the simulation method further using the 1/8-unit cell with simplified boundary conditions (**Fig. S9**). The numerically evaluated von Mises stress distributions of P- (Fig. S9a) and N-type (Fig. S9b) shell lattices using the unit cell with PBCs and the 1/8-unit cell with simplified boundary conditions show a similar pattern, with close results for the normalized Young's modulus ($E/\overline{\rho}/E_{s}$) and normalized strength ($S/\overline{\rho}/S_{y}$). Although the stress distribution of N-type lattices shows a slightly larger discrepancy (Fig. S9b), especially near the boundary edges, the numerically evaluated Young's modulus and strength using the two simulation methods remain close, with a relative deviation below 1.5%. Therefore, the simulation method using the 1/8-unit cell of periodic lattices with simplified boundary conditions is validated to maintain numerical accuracy and save the computational cost simultaneously.

**
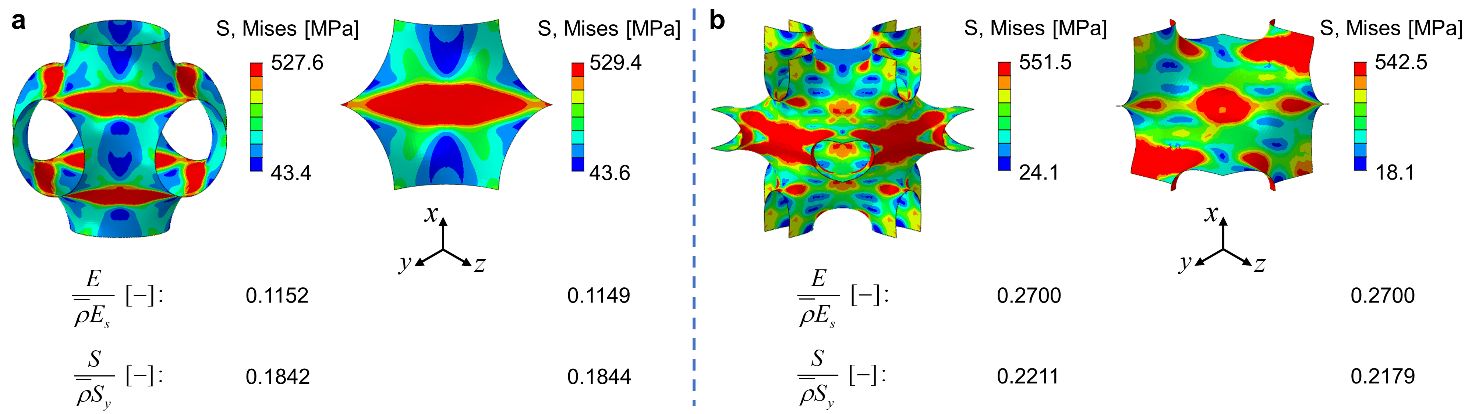
**

**Fig. S9. Comparison of numerically evaluated mechanical properties using the unit cell and 1/8-unit cell.** The von Mises stress distribution (S, Mises, under a compressive strain of 0.01), normalized Young's modulus ($E/\overline{\rho}/E_{s}$), and normalized strength ($S/\overline{\rho}/S_{y}$) of (a) P- and (b) N-type TPMS shell lattices with 1.75% RD, using the unit cell with PBCs and 1/8-unit cell with simplified boundary conditions.

# Comparison of mechanical properties of ribbed and unribbed P-type shell lattices made of other constitutive materials

The mechanical properties of three representative P-type TPMS shell lattices, including LOA-I ribbed, LOC-I ribbed, and the unribbed lattices, that are made of another constitutive material -- magnesium alloy AM60, are further compared. Within the range of finite compressive strain considered in this study, the magnesium alloy AM60 material exhibits only a slight difference in the constitutive stress-strain curves under tensile and compressive states [5]. Accordingly, the nominal stress-strain curve of the magnesium alloy AM60 obtained from uniaxial tensile tests is converted into the true stress-plastic strain curve (Fig. S10a), which is then used for the numerical simulation. The magnesium alloy AM60 material possesses a Young's modulus of *E_s_* = 44765.39 MPa, a Poisson's ratio of *ν_s_* = 0.35, and a yield strength of *S_y_* = 139.95 MPa [5]. The P-type TPMS shell lattices made of magnesium alloy AM60 are shown to undergo the yield-to-buckling failure mode transition in the 1.5%~2.0% RD range (Fig. S10b). The incorporation of LOA-I and LOC-I ribs can also enable significant strength enhancement of P-type shell lattices, especially at low RDs (Fig. S10c). The enhanced strength is attributed to rib-enabled stability enhancement via buckling suppression, as manifested by their von Mises stress distributions (Fig. S10d, under a macro stress of 0.4 MPa), validating again the rib reinforcement design strategy proposed in this study. Furthermore, given the same RD, the lattices made of magnesium alloy AM60 exhibit only slightly lower normalized strength ($S/\overline{\rho}/S_{y}$) than that of the lattices made of SS316L, with the relative deviation lower than 6.5%, indicating that changing the constitutive material from SS316L to magnesium alloy AM60 does not have a significant effect on their normalized strength.


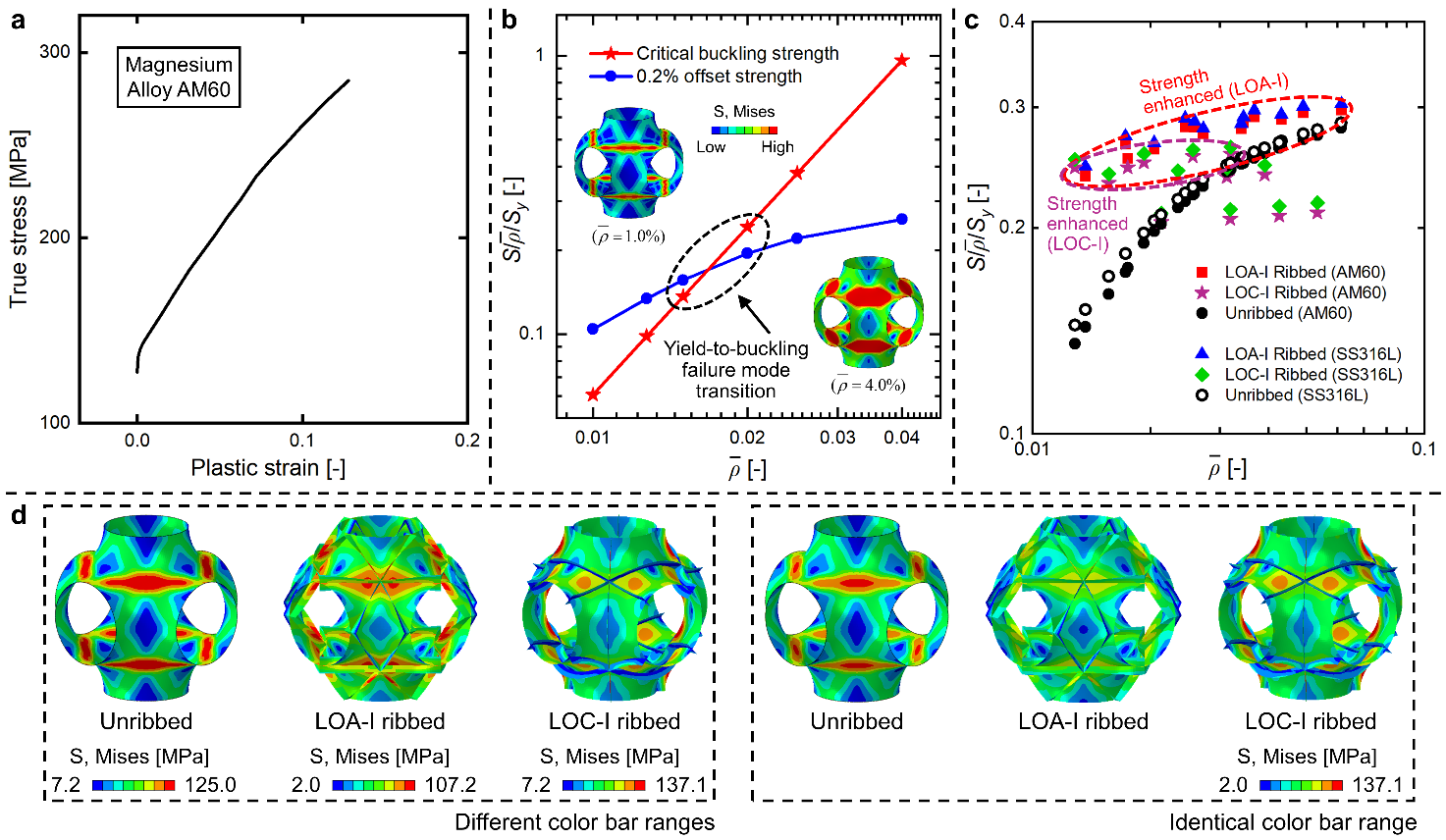


**Fig. S10. The mechanical properties and rib reinforcement effect of P-type TPMS shell lattices made of magnesium alloy AM60.** (a) The true stress versus plastic strain curve of magnesium alloy AM60, (b) the yield-to-buckling failure mode transition of P-type TPMS shell lattices made of magnesium alloy AM60, (c) the normalized strength ($S/\overline{\rho}/S_{y}$) versus RD ($\overline{\rho}$) plots of LOA-I ribbed, LOC-I ribbed, and the unribbed P-type shell lattices made of magnesium alloy AM60 (and SS316L as a comparison), and (d) their von Mises stress distributions under a macro stress of 0.4 MPa, in which the original different ranges and the unified identical range of the color bar are shown, respectively.

# Numerical results of N-type TPMS shell lattices

## Linear buckling and nonlinear static analyses of N-type TPMS shell lattices

The linear buckling and nonlinear static analyses results of N-type TPMS shell lattices with RDs ranging 1.0%~4.0% are shown in **Fig. S11**. The normalized strength versus RD plot reveals that N-type lattices also undergo the yielding-to-buckling failure mode transition in the 1.5%~2.0% RD range (Fig. S11a-b). The transition is manifested as a sudden change in the slope of the normalized strength versus RD plot in logarithmic coordinates (Fig. S11a) and as a significant difference in the deformation state and von Mises stress distribution between lattices with different RDs (Fig. S11b). The critical buckling mode reveals that the umbilical regions undergo severe out-of-plane deformations (Fig. S11c). In the nonlinear static analysis of lattices with RDs of 1.5% and lower, the umbilical regions parallel to the loading (*z*) direction undergo out-of-plane deformations and high stresses (Fig. S11b), indicating a shell buckling failure mode. In contrast, these regions are mostly under uniform compressive deformations at 2.0% and higher RDs, indicating a failure mode dominated by material yielding (Fig. S11b).


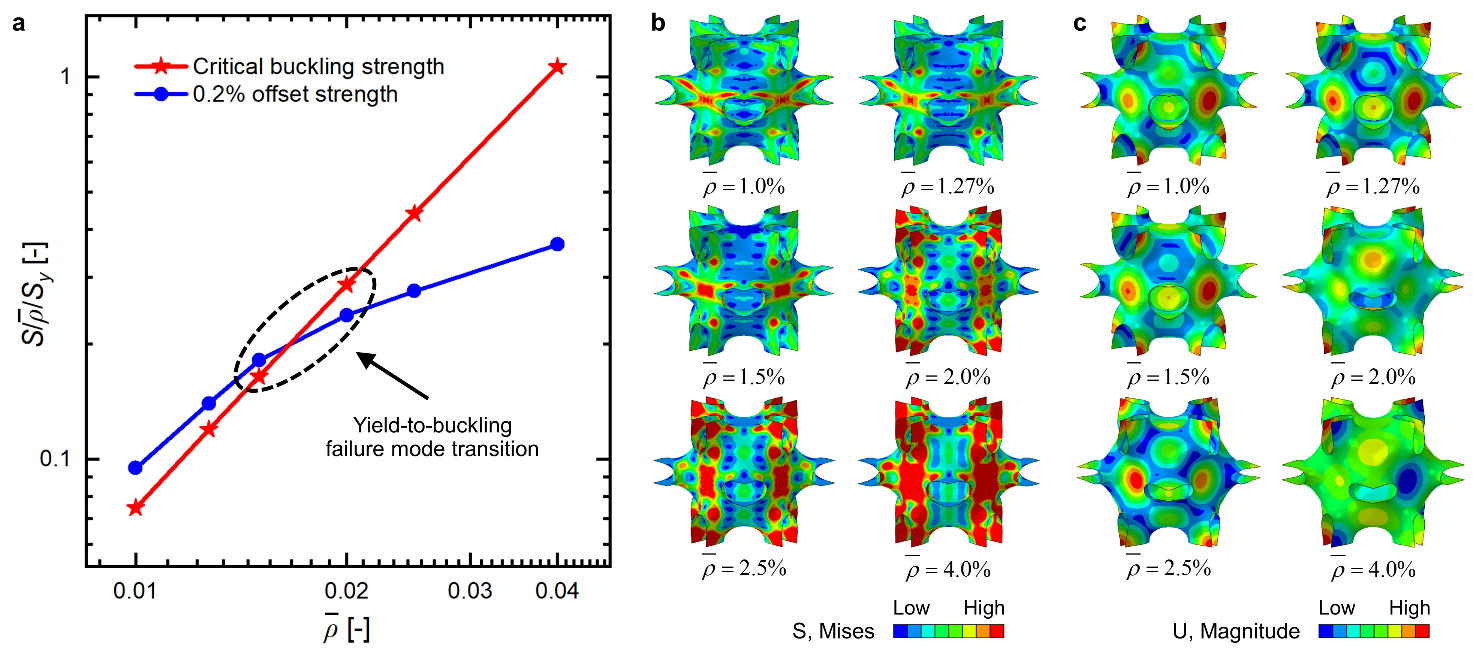


**Fig. S11. Linear buckling and nonlinear static analyses of N-type TPMS shell lattices with RDs ranging 1.0%~4.0%.** (a) Normalized strength versus RD plots of N-type shell lattices, including the normalized critical buckling strength and the normalized 0.2% offset strength. (b) The von Mises stress distributions at the 0.2% offset point and (c) the critical buckling modes of N-type shell lattices with varying RDs.

## Numerical results of ribbed N-type TPMS shell lattices

The incorporation of appropriate rib layouts was also explored for N-type TPMS shell lattices to illustrate further the generality of the proposed rib reinforcement design method. The numerically evaluated strength, stress-strain curves, and von Mises stress distributions of four representative layouts of LOA and LOC ribbed N-type shell lattices with varying RDs and their unribbed counterparts with equal RDs are shown in **Fig. S12**a-p, respectively. By incorporating ribs to suppress the shell buckling deformation that mainly occurs in the umbilical region, the strength of ultralight N-type shell lattices can be significantly improved. Among the four layouts of LOA ribbed lattices, the incorporation of LOA-r6 (Fig. S12c and g) and LOA-r7 (Fig. S12d and h) ribs can enhance the strength of N-type shell lattices, while incorporating LOA-r1 (Fig. S12a and e) and LOA-r3 (Fig. S12b and f) ribs results in slight improvement or even a reduction in strength. The LOA-r7 (Fig. S12d and h) ribs directly pass through the umbilical region of N-type shell lattices that underwent the maximum displacement in the buckling mode, thus leading to the highest strength enhancement among the four layouts of LOA ribbed lattices. In comparison, the LOA-r6 (Fig. S12c and g) rib that passes through the umbilical region with smaller deformations in the buckling mode, leading to lower strength enhancement. In particular, the LOA-r7 ribbed lattice can achieve 69.5% strength enhancement at 1.36% RD, while the LOA-r6 ribbed lattice achieves 18.8% enhancement at 1.24% RD.

Among the four layouts of LOC ribbed lattices, incorporating LOC-r1 (Fig. S12i and m), LOC-r6 (Fig. S12k and o), and LOC-r7 (Fig. S12l and p) ribs can enhance the strength of N-type shell lattices, while the incorporation of LOC-r3 (Fig. S12j and n) ribs tends to decrease the strength. The LOC-r1 (Fig. S12i and m) ribs, which directly pass through the umbilical region with significant buckling deformations, result in the highest magnitude of strength enhancement. The LOC-r7 (Fig. S12l and p) rib also crosses through the umbilical region and can lead to the second-highest strength enhancement. In comparison, the strength enhancement from incorporating the LOA-r6 (Fig. S12k and o) rib is lower, whereas incorporating the LOC-r3 (Fig. S12j and n) rib reduces the strength. More specifically, the LOC-r1 (Fig. S12i) ribbed lattice can achieve 82.3% strength enhancement at 1.20% RD, the LOC-r7 (Fig. S12l) ribbed lattice achieves 50.6% enhancement at 1.14% RD, and the LOC-r6 (Fig. S12k) ribbed lattice achieves 13.6% enhancement at 1.23% RD. The raw data of the normalized strength of the 7 layouts of LOA and LOC ribbed lattices are listed in the Supplementary File "Original Data.xlsx".


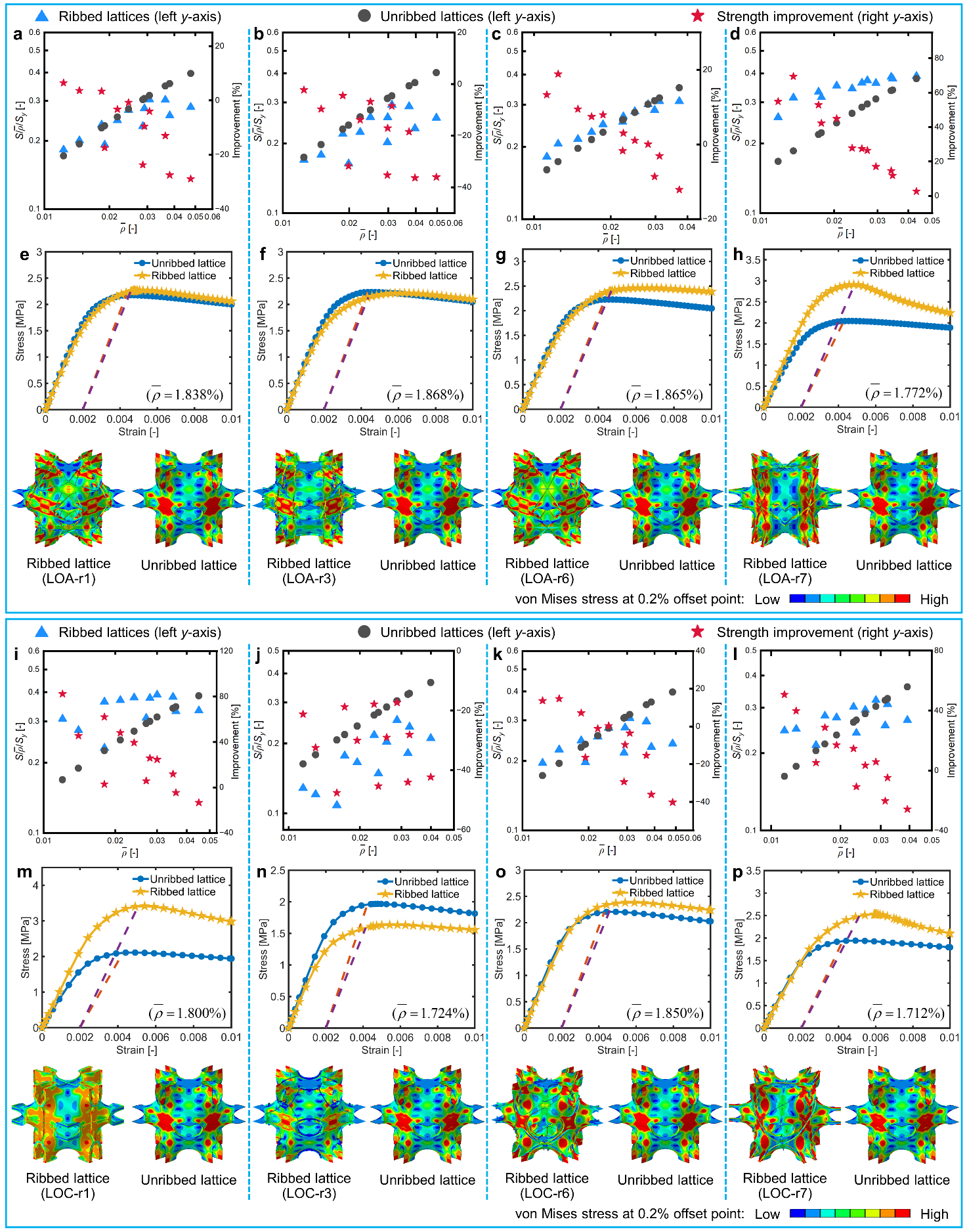


**Fig. S12. Simulation results of four representative layouts of LOA and LOC ribbed N-type TPMS shell lattices and their unribbed counterparts with equal RDs.** The normalized strength versus RD plots of (a) LOA-r1, (b) LOA-r3, (c) LOA-r6, and (d) LOA-r7 ribbed lattices and their unribbed counterparts, and (e-h) their stress-strain curves and von Mises stress distributions at the 0.2% offset point, as compared to their unribbed counterparts. The normalized strength versus RD plots of (i) LOC-r1, (j) LOC-r3, (k) LOC-r6, and (l) LOC-r7 ribbed lattices and their unribbed counterparts, and (m-p) their stress-strain curves and von Mises stress distributions at the 0.2% offset point, as compared to their unribbed counterparts.

# Comparison of strength with existing ultralight and hierarchical shell lattices

The normalized strength ($S/\overline{\rho}/S_{y}$) of the ribbed P- and N-type TPMS shell lattices proposed in this study are further compared to that of their ultralight [6-8] and hierarchical [9-11] counterparts in existing studies (**Fig. S13**). The rib-reinforced P- and N-type ultralight shell lattices both outperform their unribbed counterparts in existing studies in strength significantly [6-8], and more strikingly, the strength of ribbed P-type shell lattices can be improved to be close to that of N-type shell lattices. In comparison, the hierarchical TPMS shell lattices in existing studies generally exhibit lower strength than the ribbed lattices with equal RDs [9-11]. Therefore, as compared to the incorporation of hierarchical micro-architecutures, the proposed rib reinforcement design strategy can enable more effective buckling suppression and result in a greater magnitude of strength enhancement for ultralight shell lattices.


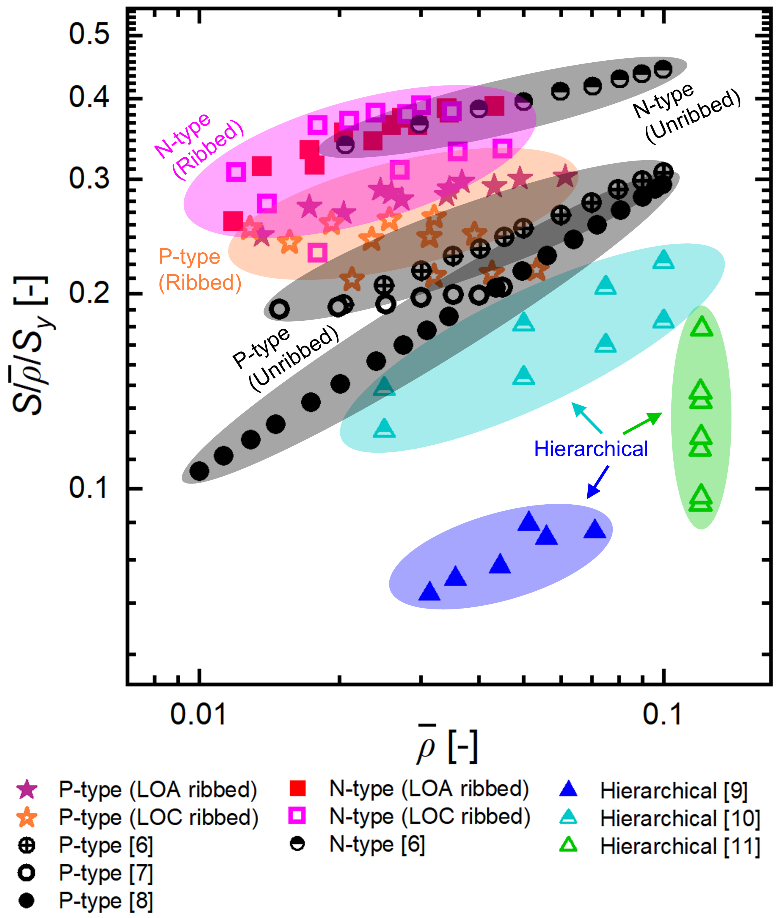


**Fig. S13. Comparison of the normalized strength ($S/\overline{\rho}/S_{y}$) of the proposed rib-reinforced P- and N-type TPMS shell lattices with their ultralight and hierarchical counterparts in existing studies [6-11].**

# Constitutive properties of stainless steel 316 L (SS316L) constituent materials

The uniaxial tensile test of standard tensile samples made by micro laser powder bed fusion (µLPBF) was conducted to determine the stress-strain curve of the constitutive SS316L materials, based on the ASTM E8/E8M standard [2]. The test reveals the Young's modulus (*E_s_* = 189310.0 MPa), Poisson's ratio (*ν_s_* = 0.3), and uniaxial yield strength (*S_y_* = 520.0 MPa) of the SS316L materials, which, together with their true stress versus plastic strain curve (**Fig. S14**), were utilized in the nonlinear simulation of the ribbed and unribbed shell lattices.


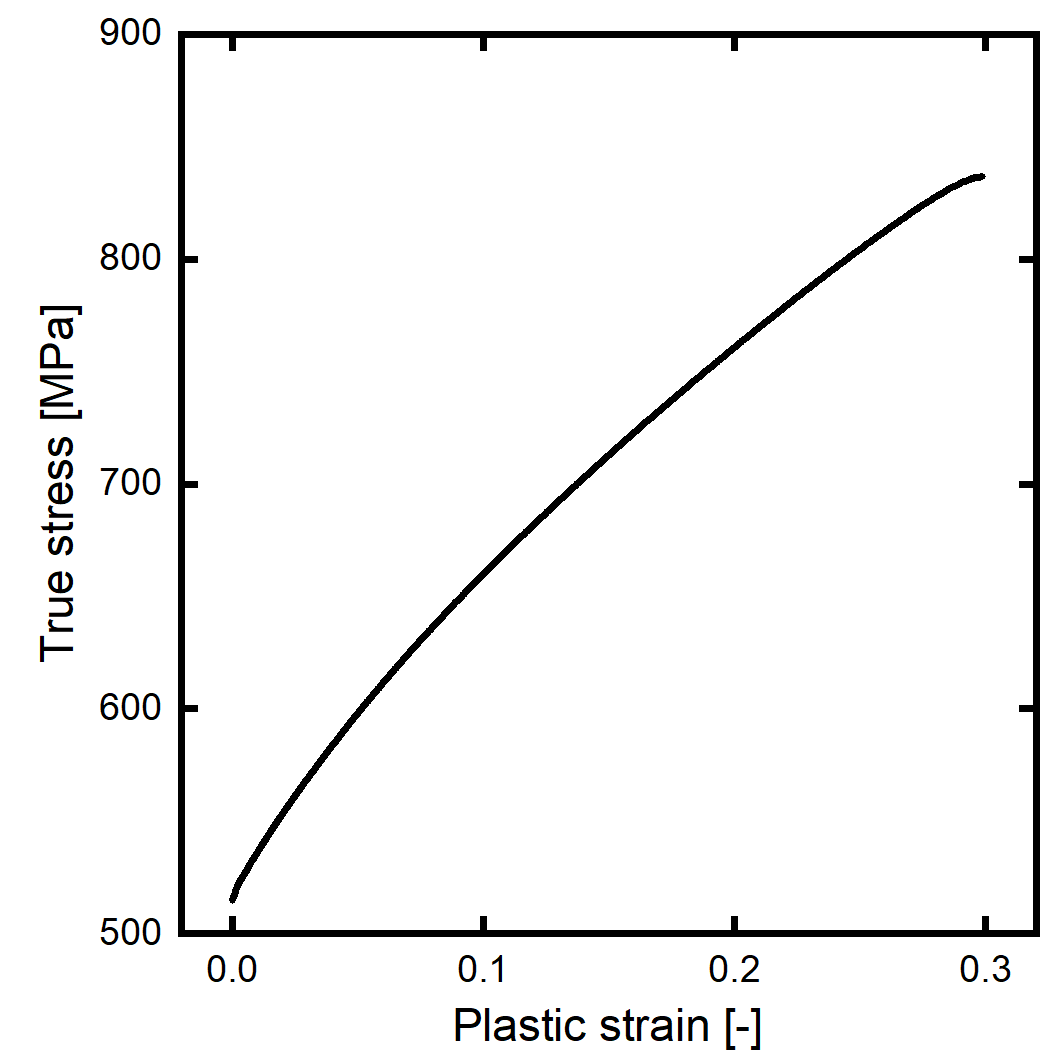


**Fig. S14. The true stress versus plastic strain curve of the constitutive SS316L materials.**

# Experimental methods and data of ribbed and unribbed P-type shell lattices

In the experimental section, the Young's moduli of the lattices were evaluated as the average slope of the linear stage of 3 unload curves from the load-unload repetitive compression tests, and the effect of machine stiffness was eliminated to avoid underestimating the Young's moduli of the samples [3]. The 0.2% offset strength was evaluated as the strength of samples in large-strain compression tests under a nominal strain rate of 0.001 s^-1^ [2, 6]. The NPS ($\sigma_{p}$), namely normalized plateau stress, was evaluated as the average stress (within 0.2~0.4 strain range) divided by the RD [3]:

$$\sigma_{p}=\frac{5}{\overline{\rho}}\int_{0.2}^{0.4} \sigma(\varepsilon)d\varepsilon.$$

The energy absorption efficiency ($\eta$) of the samples was determined as [3]:

$$\eta=\frac{1}{\sigma(\varepsilon)}\int_{0}^{\varepsilon} \sigma(e)de,$$

in which the densification strain ($\varepsilon_{d}$) was extracted as the strain in which $\eta$ attains its maximum. Based on this, the specific energy absorption (SEA, $\psi$) of the samples was evaluated as [3]:

$$\psi=\frac{1}{\overline{\rho}}\int_{0}^{\varepsilon_{d}} \sigma(\varepsilon)d\varepsilon.$$

The experimentally measured RDs ($\overline{\rho}$), normalized Young's moduli ($E/\overline{\rho}/E_{s}$), normalized strength ($S/\overline{\rho}/S_{y}$), normalized plateau stresses (NPSs, $\sigma_{p}$), and specific energy absorption (SEA, $\psi$) of the samples are shown in **Fig. S15**. The RDs of LOA ribbed, LOC ribbed, and the unribbed samples were measured as 2.86%$\pm$0.03%, 2.58%$\pm$0.03%, and 2.39%$\pm$0.04%, respectively (Fig. S15a), higher than the as-designed value (2.03%). The normalized Young's moduli were measured as 6.05$\times$10^-2^$\pm$1.78$\times$10^-3^, 4.69$\times$10^-2^$\pm$1.39$\times$10^-3^, and 2.97$\times$10^-2^$\pm$1.44$\times$10^-3^, respectively (Fig. S15b), in which the machine stiffness was eliminated to avoid stiffness underestimation [3]. Therefore, incorporating LOA and LOC ribs can enhance the stiffness of P-type shell lattices by 103.70% and 57.91%, respectively. The normalized strength of LOA and LOC ribbed samples was measured as 0.136$\pm$0.006 and 0.104$\pm$0.003, which were 94.29% and 48.57% higher than that of the unribbed samples (0.070$\pm$0.003), respectively (Fig. S15c). The NPSs of LOA ribbed, LOC ribbed, and the unribbed samples were measured as 59.06$\pm$2.19 MPa, 39.21$\pm$3.07 MPa, and 40.22$\pm$1.89 MPa (Fig. S15d), and their SEAs were measured as 35.24$\pm$3.87 J/cm^3^, 21.81$\pm$3.43 J/cm^3^, and 19.21$\pm$3.10 J/cm^3^ (Fig. S15e), respectively. In other words, the LOA ribbed samples exhibit 46.84% and 83.45% higher NPS and SEA than the unribbed samples, while the NPS and SEA of LOC ribbed samples do not change too much as compared to the unribbed samples. The mechanical properties of the three repetitive samples within each group exhibit a low magnitude of relative standard deviation, validating the repeatability of experimental data based on the µLPBF fabricated high-fidelity samples. Overall, the experimental results demonstrate the effectiveness of the proposed design of ribbed shell lattices for strength enhancement, and further indicate that other mechanical properties, including Young's modulus, plateau stress, and specific energy absorption, can be simultaneously improved, especially for the LOA ribbed lattices.


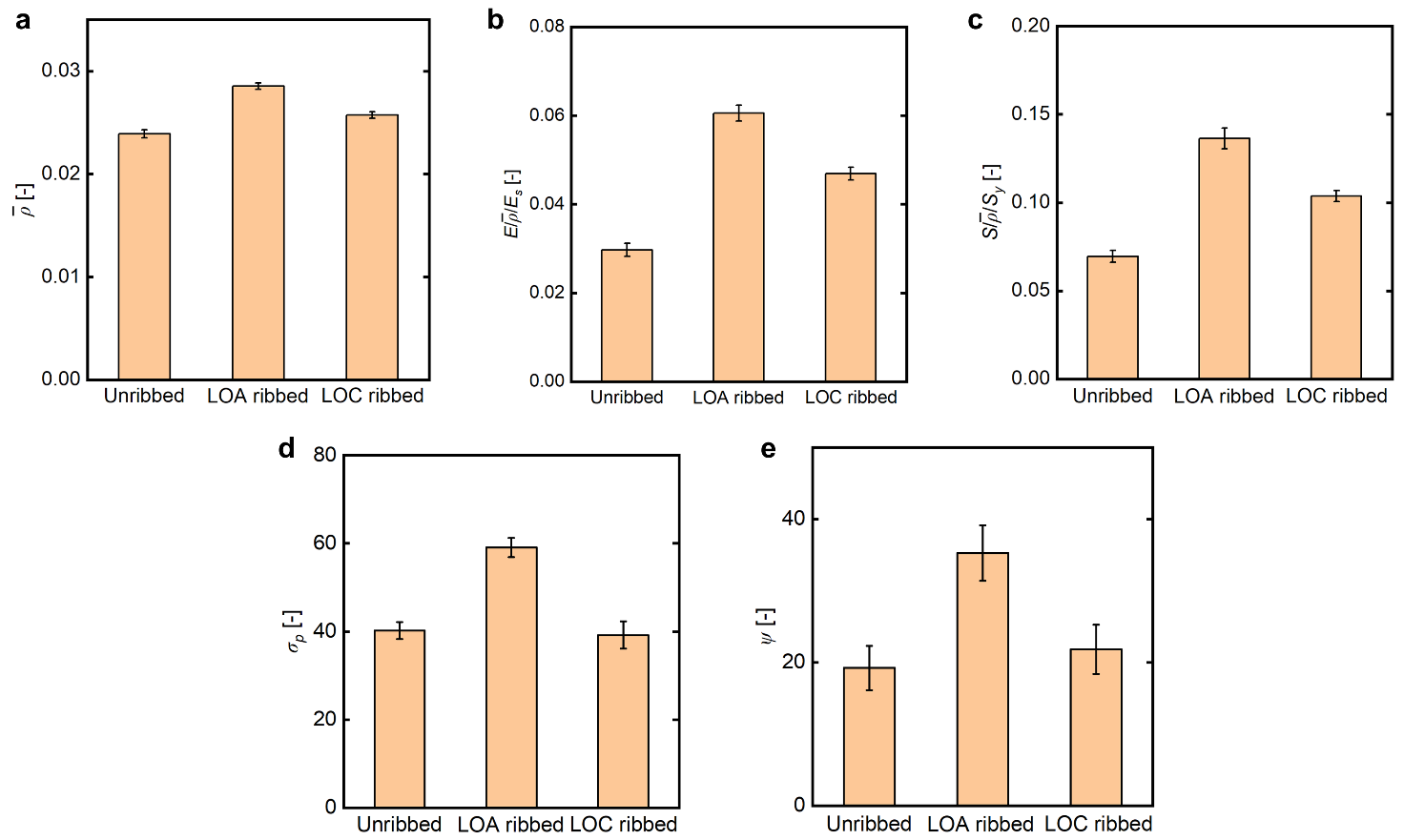


**Fig. S15. Experimental data of the unribbed, LOA ribbed, and LOC ribbed samples of P-type TPMS shell lattices.** The experimentally measured (a) RDs ($\overline{\rho}$), (b) normalized Young's moduli ($E/\overline{\rho}/E_{s}$), (c) normalized strength ($S/\overline{\rho}/S_{y}$), (d) NPSs ($\sigma_{p}$), and (e) SEAs ($\psi$) of the unribbed, LOA ribbed, and LOC ribbed samples.

# Sensitivity discussion on the effect of discrepancy in as-designed and as-built wall thicknesses on the mechanical properties of samples

The experimentally measured average RDs ($\overline{\rho}$) of LOA ribbed, LOC ribbed, and the unribbed samples are 2.86%$\pm$0.03%, 2.58%$\pm$0.03%, and 2.39%$\pm$0.04%, which are 40.89%, 27.09%, and 17.73% higher than the as-designed RD (2.03%), respectively. The discrepancy is primarily attributed to the larger wall thicknesses of the as-fabricated samples than those of the as-designed geometries, especially arising from the inevitable surface roughness, and the thickness deviations of ribs are generally greater than those of shells (Fig. 4a). The micro-CT characterized wall thickness (*δ*) distributions of the as-fabricated samples demonstrate that the average wall thicknesses of LOA ribbed, LOC ribbed, and the unribbed samples are 106.53, 96.20, and 85.83 µm, respectively (Fig. 4a). As a comparison, the as-designed average wall thicknesses of LOA ribbed, LOC ribbed, and the unribbed samples are 75.39, 75.57, and 70 µm, respectively.

To achieve a closer match between the as-designed and as-built wall thicknesses, a common approach is to evaluate the effective wall thickness (*δ*_eff_) as the difference between the as-measured nominal wall thickness (*δ*) and twice the average surface roughness (*R_a_*) [12, 13], namely *δ*_eff_ = *δ* – 2*R_a_*. The average surface roughness is measured as *R_a_* ≈ 5 µm in our prior study [14], based on which the effective wall thicknesses (*δ*_eff_) of LOA ribbed, LOC ribbed, and the unribbed samples are evaluated as 96.53 µm, 86.20 µm, and 75.83 µm, respectively. Accordingly, the effective RDs ($\overline{\rho}_{eff}$) of the samples are calculated as 2.59%, 2.31%, and 2.11%, respectively, which are closer to the as-designed RD (2.03%). The remaining discrepancies are mainly due to other manufacturing defects that induce deviations in as-built wall thicknesses from the as-designed value, such as the wall thickness distribution related to the inclination angle [15]. These discrepancies can be further mitigated through compensation design in the manufacturing process and will be explored in detail in our subsequent studies. Given the uncertainty in the measurement process of average surface roughness, the mechanical properties of samples, including Young's modulus, strength, plateau stress, and energy absorption, are normalized against the nominal RD ($\overline{\rho}$), rather than the effective RD ($\overline{\rho}_{eff}$), as listed in the main text. Overall, the discrepancy in the as-designed and as-built wall thicknesses and RDs does induce a certain degree of deviation in the mechanical properties of samples, while the deviation is within an acceptable range, and the experimental results do indeed validate the rib-enabled strength enhancement of ultralight shell lattices.

**References**

[1] E. Ventsel, T. Krauthammer, E. Carrera, Thin plates and shells: theory, analysis, and applications, Appl. Mech. Rev. 55(4) (2002) B72-B73.

[2] J. Ding, Q. Ma, X. Li, L. Zhang, H. Yang, S. Qu, M.Y. Wang, W. Zhai, H. Gao, X. Song, Imperfection-Enabled Strengthening of Ultra-Lightweight Lattice Materials, Adv. Sci. (2024) 2402727.

[3] Q. Ma, L. Zhang, J. Ding, S. Qu, J. Fu, M. Zhou, M.W. Fu, X. Song, M.Y. Wang, Elastically-isotropic open-cell minimal surface shell lattices with superior stiffness via variable thickness design, Addit. Manuf. 47 (2021) 102293.

[4] S. Li, Boundary conditions for unit cells from periodic microstructures and their implications, Compos. Sci. Technol. 68(9) (2008) 1962-1974.

[5] U. Ali, Numerical modeling of failure in magnesium alloys under axial compression and bending for crashworthiness applications, University of Waterloo, 2012.

[6] Y. Wang, X. Zhang, Z. Li, H. Gao, X. Li, Achieving the theoretical limit of strength in shell-based carbon nanolattices, Proc. Natl. Acad. Sci. U.S.A. 119(34) (2022) e2119536119.

[7] S.C. Han, K. Kang, Another stretching-dominated micro-architectured material, shellular, Mater. Today 31 (2019) 31-38.

[8] X. Guo, J. Ding, X. Li, S. Qu, X. Song, J.Y.H. Fuh, W.F. Lu, W. Zhai, Enhancement in the mechanical behaviour of a Schwarz Primitive periodic minimal surface lattice structure design, Int. J. Mech. Sci. 216 (2022) 106977.

[9] L. Zhang, Z. Hu, M.Y. Wang, S. Feih, Hierarchical sheet triply periodic minimal surface lattices: Design, geometric and mechanical performance, Mater. Des. 209 (2021) 109931.

[10] H. Liu, W.W.S. Ma, J. Ding, S. Qu, R. Li, Q. Ge, M.Y. Wang, X. Song, Hierarchical triply periodic minimal surface shell lattices with superior isotropic elasticity: Design guidelines, fabrication, and validation, Addit. Manuf. 94 (2024) 104451.

[11] R. Li, W.W.S. Ma, T. Niu, H. Liu, J. Ding, X. Song, Stiffness and strength enhancement of hierarchical TPMS-based shell lattices via inter-level conformal design, Addit. Manuf. (2025) 104802.

[12] L. Zhang, S. Feih, S. Daynes, S. Chang, M.Y. Wang, J. Wei, W.F. Lu, Energy absorption characteristics of metallic triply periodic minimal surface sheet structures under compressive loading, Addit. Manuf. 23 (2018) 505-515.

[13] J. Fu, J. Ding, S. Qu, L. Zhang, M.Y. Wang, M.W. Fu, X. Song, Improved light-weighting potential of SS316L triply periodic minimal surface shell lattices by micro laser powder bed fusion, Mater. Des. (2022) 111018.

[14] L. Zhang, M. Cang, J. Ding, W.W.S. Ma, X. Zhu, Y. Lu, X. Song, H. Cui, M.Y. Wang, Metallic perforated plate lattices with superior buckling strength, Mater. Des. (2024) 113544.

[15] J. Ding, S. Qu, L. Zhang, M.Y. Wang, X. Song, Geometric deviation and compensation for thin-walled shell lattice structures fabricated by high precision laser powder bed fusion, Addit. Manuf. 58 (2022) 103061.
